# Individual diet variability shapes the architecture of Antarctic benthic food webs

**DOI:** 10.1038/s41598-024-62644-5

**Published:** 2024-05-29

**Authors:** Simona Sporta Caputi, Jerzy Piotr Kabala, Loreto Rossi, Giulio Careddu, Edoardo Calizza, Matteo Ventura, Maria Letizia Costantini

**Affiliations:** 1 Department of Environmental Biology, Sapienza University of Rome, Via Dei Sardi 70, 00185 Rome, Italy; 2 CoNISMa, National Inter-University Consortium for Marine Sciences, Piazzale Flaminio 9, 00196 Rome, Italy

**Keywords:** Ecology, Biodiversity, Climate-change ecology, Community ecology, Conservation biology, Ecological modelling, Ecological networks, Ecosystem ecology, Stable isotope analysis, Ecology, Food webs

## Abstract

Antarctic biodiversity is affected by seasonal sea-ice dynamics driving basal resource availability. To (1) determine the role of intraspecific dietary variability in structuring benthic food webs sustaining Antarctic biodiversity, and (2) understand how food webs and the position of topologically central species vary with sea-ice cover, single benthic individuals’ diets were studied by isotopic analysis before sea-ice breakup and afterwards. Isotopic trophospecies (or Isotopic Trophic Units) were investigated and food webs reconstructed using Bayesian Mixing Models. As nodes, these webs used either ITUs regardless of their taxonomic membership (ITU-webs) or ITUs assigned to species (population-webs). Both were compared to taxonomic-webs based on taxa and their mean isotopic values. Higher resource availability after sea-ice breakup led to simpler community structure, with lower connectance and linkage density. Intra-population diet variability and compartmentalisation were crucial in determining community structure, showing population-webs to be more complex, stable and robust to biodiversity loss than taxonomic-webs. The *core* web, representing the minimal community ‘skeleton’ that expands opportunistically while maintaining web stability with changing resource availability, was also identified. Central nodes included the sea-urchin *Sterechinus neumayeri* and the bivalve *Adamussium colbecki*, whose diet is described in unprecedented detail. The *core* web, compartmentalisation and topologically central nodes represent crucial factors underlying Antarctica’s rich benthic food web persistence.

## Introduction

The ability of a biotic community to resist or adapt to external pressures is closely related to the level of web complexity, which depends on predictable energy supply^1–10^. Climate-induced environmental changes could lead to a mismatch between food supply and demand, thus altering the food web structure^11–13^.

In Antarctica, the rich marine communities, which are highly adapted to seasonal variations in sea ice^10,14–18^, are increasingly threatened by environmental modifications. These changes are primarily due to climate change, which is leading, in addition to increasing temperatures, to variation in sea-ice extent, light regimes and nutrient availability for primary producers^15,19,20^. Resource availability for Antarctic communities depends on seasonal cycles of sea ice^11,12,16,21–24^, to which the other factors are closely linked. Hence, studying food web structures under different sea-ice conditions increases our understanding of the key mechanisms underlying the persistence of complex communities and their response to environmental variation including climate change^17,25,26^.

Reconstructing the food webs of complex communities presents challenges^9,27^. These include high biodiversity coupled with trophic generalisation and the wide range of exploitable food resources. Further, high variability in resource use by consumers, even within populations, and uncertainty about their taxonomic identity, can make it harder to identify the trophic links between species^12,13,28–30^. In addition, species’ functional traits such as the variable feeding behaviour of individuals, which determine species’ responses to changes in environmental conditions^11,31–33^, can influence community stability^9,30,31,33,34^. However, their role in determining food web structure is still uncertain or completely unknown^9,33,35–37^. With these uncertainties, the persistence of complex communities when some theory suggests such systems will be unstable remains a paradox^3,38,39^. This challenges our ability to predict their response to current and future environmental change^40^.

The development of techniques and methods based on quantitative approaches has led to increased knowledge of the structure and metrics of food webs^27,41^. However, despite the importance of developing a detailed but generalisable food web model, serious difficulties remain in reconstructing food webs that include the main energy pathways across levels of biological organisation from individuals to communities^9,11^. Stable isotope analysis of carbon (δ^13^C) and nitrogen (δ^15^N) are powerful tools that make possible integration of information across multiple levels of biological organisation (i.e. from single individuals to the entire food web) and at meaningful timescales^42,43^. Stable isotopes are thus a fundamental tool for understanding the ecological mechanisms underlying food web reorganisation under conditions of environmental change^44^. δ^13^C values can vary among primary producers, making it possible to detect and quantify the distinct contribution of each carbon source to populations’ diets and hence food webs^45–49^. δ^15^N values progressively increase with trophic level, making it possible to obtain information on the trophic position of individuals or species in the web^47,50^. Using individual δ^13^C and δ^15^N values, Rossi et al.^9^ proposed a new approach to food web reconstruction based on Isotopic Trophic Units (ITUs). ITUs were defined as either single individuals or small groups of individuals having similar δ^13^C and δ^15^N values and thus occupying the same position in the δ^13^C–δ^15^N biplot^29,51,52^. Based on ITUs, complex food webs of medium-depth (from 21 to 240 m) Antarctic benthic communities in Terra Nova Bay (Ross Sea) were reconstructed in great detail, and their spatial variation under varying degrees of sea-ice persistence was described. When a classic approach based on average population isotope values was applied to food web reconstruction in shallower waters of the same area^10^, the links were less detailed and the linkage density was lower than what was observed by Rossi et al.^9^. The differences may be ascribed to the different approaches to web reconstruction, which could thus lead to biased conclusions regarding web structure and stability.

We investigated food-web reorganisation before and after sea-ice breakup in the shallow waters of Terra Nova Bay at different scales of biological aggregation (i.e. from individuals to taxa and communities). The goal was to gain greater insight into the combined role of individuals’ diets and changing ice-related energy supply in shaping Antarctic benthic biodiversity and determining its resilience, stability and robustness to species loss.

The principal aim of this study was to increase our understanding of the mechanisms that allow communities adapted to extremely variable environments to be highly complex yet resilient. This entailed investigating intrapopulation dietary variability as a central functional trait affecting food web structure. In agreement with *Optimal foraging* theory^53,54^, we tested the hypothesis that high food availability after sea-ice breakup leads to a simplification of food webs due to specialisation and diversification of individuals’ diets. Given the persistence of the Antarctic community, we also hypothesised that there exists a *core* web representing the invariant skeleton of community structure that remains impervious to changes in resource availability, thus playing a crucial role in community stability. Under this premise, the study addressed three specific goals. First, we reconstructed and compared food web structure and metrics before and immediately after sea-ice breakup (i.e. Low vs. High food availability^14,17,21,24,55–58^). Second, we compared webs reconstructed using three different approaches: a. *ITU-webs* (following^9^), in which the nodes were single ITUs regardless of species; b. *population-webs*, in which nodes were species. In this case, each species’ trophic links were derived from the trophic links of the ITUs belonging to that species, thus accounting for ‘intraspecific’ variability; and c. *taxonomic-webs*, reconstructed using each species’ average isotope values, without taking account of ITUs (according to^10^). Third, we identified the core food web whose links and nodes remain unchanged. Comparisons were extended from the whole web to species level, by focusing on key Antarctic species in terms of abundance and distribution (the scallop *Adamussium colbecki*, the sea-urchin *Sterechinus neumayeri* and the sea star *Odontaster validus*^59,60^).

## Results

### Biodiversity and food webs

**Figure 1:**
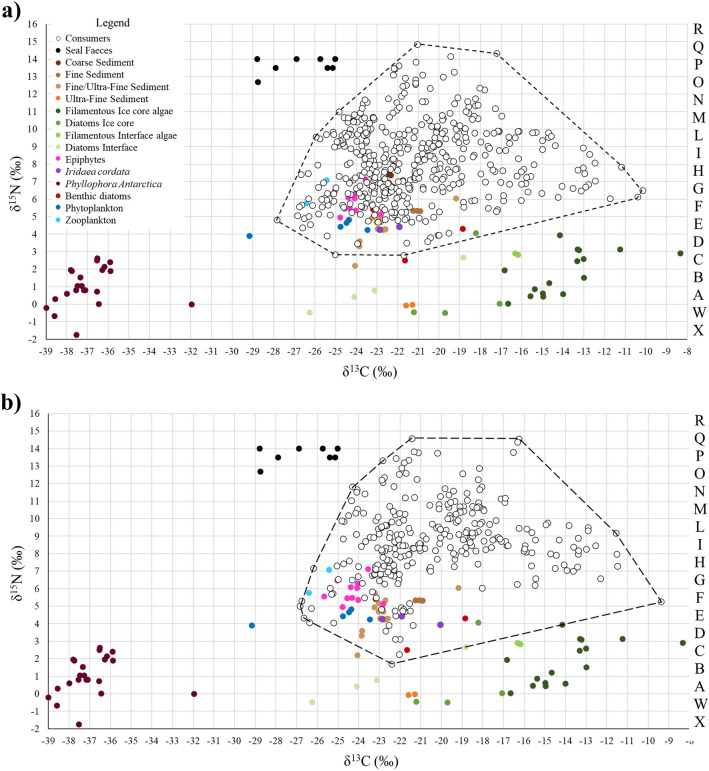
Isotopic biplot of the benthic community (**a**) before (November–December 2016) and (**b**) after (January–February 2017) sea-ice breakup in Tethys Bay (Terra Nova Bay, Ross Sea). Each point represents a specimen. Full symbols represent basal food sources, empty symbols represent consumers. The isotopic space is divided into ITUs (Isotopic Trophic Units, i.e. 1 × 1‰ intervals of δ^15^N and δ^13^C) in accordance with Rossi et al.^9^. Dashed-red line polygons delimit the convex hull (Total Area) of consumers. For the detailed list of species contributing to each sediment fraction, refer to Tables S1 and S2.

Zoobenthic diversity was lower after sea-ice breakup: 59 versus 36 taxa and 109 versus 87 ITUs (before vs. after; Fig. 1, Tables S1, S2). Samples of zoobenthos included the zooplanktonic *Limacina helicina*, its predator *Clione limacina antarctica* and benthic calanoid copepods that were found on the bottom. Since most of the zoobenthos was identified at the species level (Table S1), for ease of reading, the terms 'species' and 'intraspecific variability' are generally used in the text to indicate 'taxon' and ‘inter-individual variability', respectively. The mean number of ITUs per species did not differ between the two periods (4.8 ± 0. 4 before and 4.8 ± 0.7 after sea-ice breakup; Mann Whitney test, U: 1838, n.s.), while the mean number of species per ITU was lower after sea-ice breakup (2.5 ± 0.2 vs. 1.8 ± 0.1 species/ITU; Mann Whitney test, U: 7227.50, *p* < 0.05).

Although the number of nodes was higher when taking account of ITUs, node number was unrelated to the proportion of basal, intermediate and top nodes in the webs, the number of trophic links, or the web metrics mentioned below (R^2^ < 0.55, *p* always n.s. N = 6).

**Figure 2:**
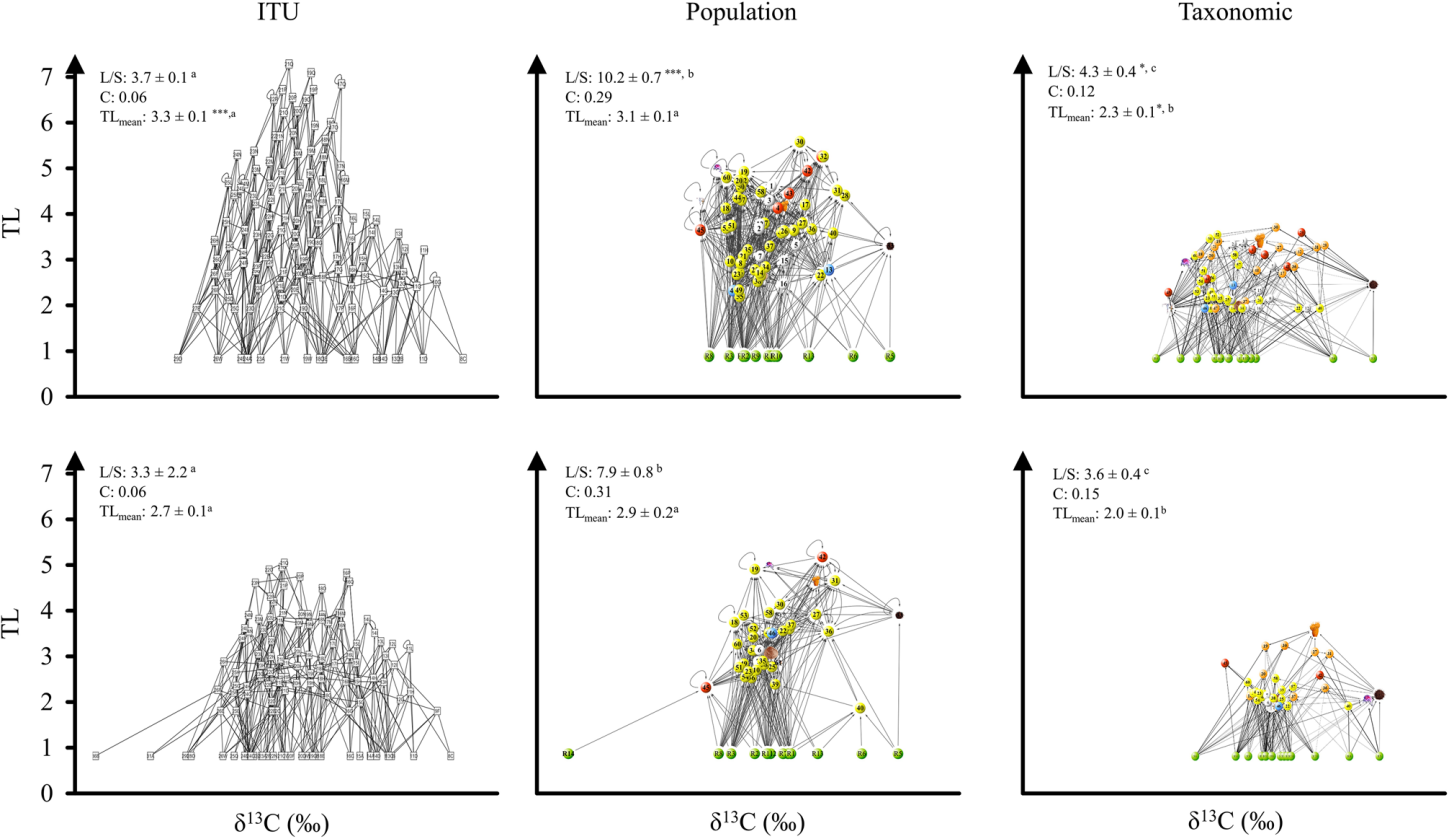
Benthic food web structure in Tethys Bay (Terra Nova Bay, Ross Sea). Top: before sea-ice breakup; bottom: after sea-ice breakup. Each node represents one ITU in the ITU-webs and one species (or a basal resource) in population-webs and taxonomic-webs. Each arrow points from a resource to its consumer, thickness being proportional to linkage strength. Each node is positioned with respect to its δ^13^C value and Trophic Level (TL). Basal resources are shown in green. Superscript asterisks (*) indicate significant differences between before and after sea-ice breakup, one, two and three asterisks indicating *p* < 0.05, *p* < 0.01 and *p* < 0.001 respectively. Superscript letters (^a^,^b^,^c^) indicate differences between food web types. For statistical tests, see “Materials and methods” section. The food web graphs were developed using Cytoscape software. For the list of species, refer to Table S1.

**Figure 3:**
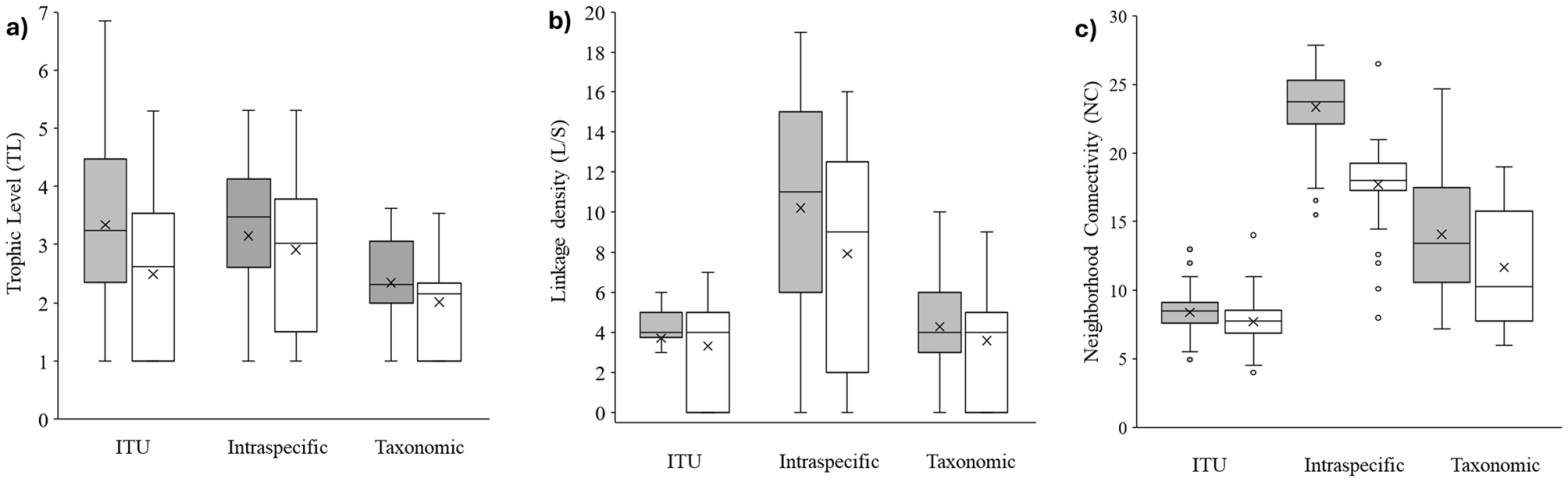
Box plot of the distribution of Trophic Level (TL), Linkage Density (L/S) and Neighbourhood Connectivity (NC) for each food web type (ITU, population and taxonomic) before (grey) and after (white) sea-ice breakup. For each panel, the horizontal line represents the median of the distribution, and the “x” symbol represents the mean. The box includes 50% of the data while the whiskers mark the highest and lowest values including 95% of the distribution.

**Figure 4:**
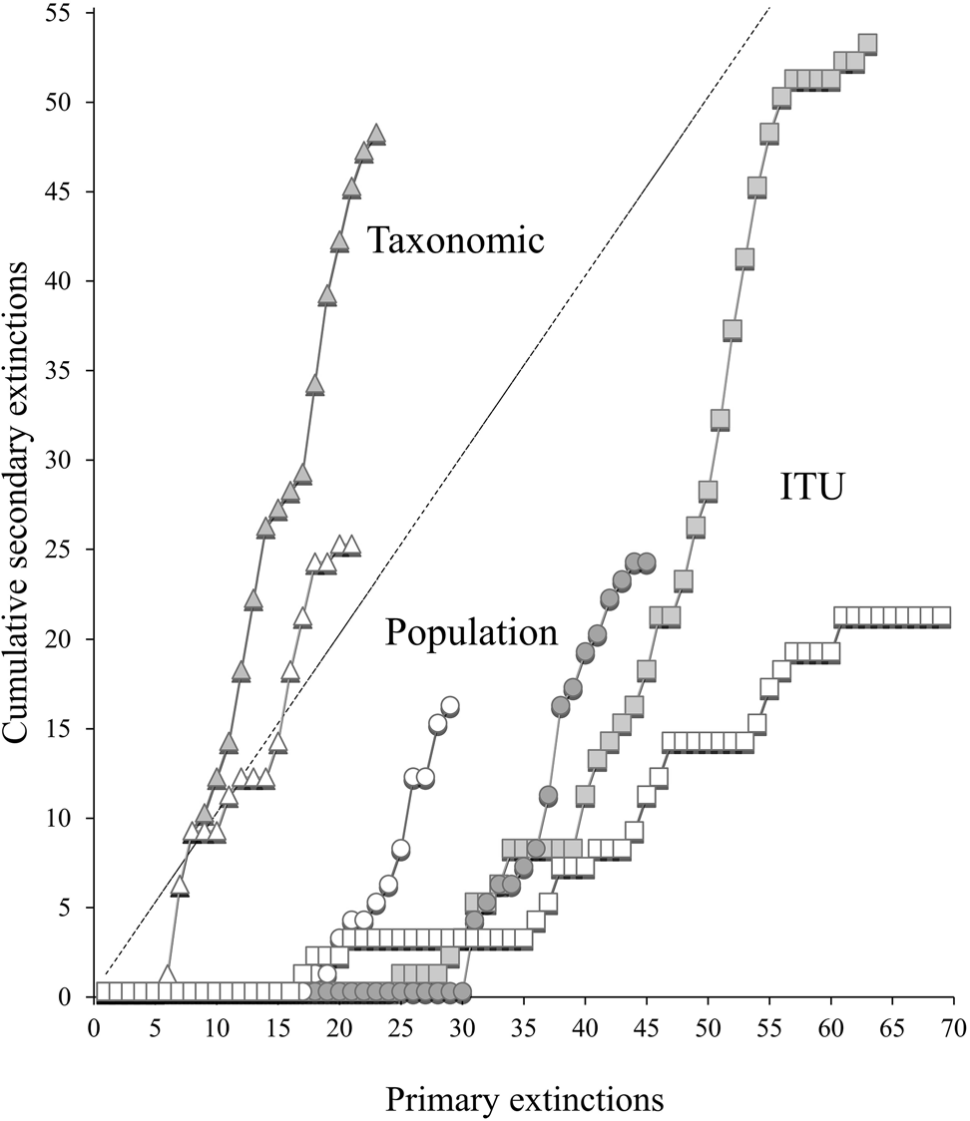
Robustness trend (primary extinction against cumulative secondary extinction) for ITU-webs (squares), population-webs (circles) and taxonomic-webs (triangles) before (grey) and after (white) sea-ice breakup.

Web topology and metrics before sea-ice breakup differed from those after and among food web types (ITU-webs, population-webs and taxonomic-webs; Tables 1, 2, Fig. 2). Trophic Level (TL) was always lower in taxonomic-webs than the other web types and was generally lower after sea-ice breakup (Table 1, Fig. 3). ITU-webs were less complex (i.e., less connected) and compartmentalised than the other webs, and also had the lowest Neighbourhood Connectivity (NC) and potential competition. By contrast, population-webs were the most complex and compartmentalised of all types of webs, with the highest connectance, linkage density (L/S), Neighbourhood Connectivity (NC), and potential competition (Table 1, Fig. 3). The last three metrics were lower after sea-ice breakup (Table 1, Fig. 3). Robustness to secondary loss of nodes was greatest in population-webs (ANCOVA test, F: 52.29, *p* < 0.001; Table 1, Fig. 4).

**Table 1:** Food web metrics before and after sea-ice breakup for ITU-webs (Trophic-functional variability), population-webs (intrapopulation variability) and taxonomic-webs (mean population variability).

Food web metrics	ITU-based approach (trophic-functional variability)		Population-based approach (intrapopulation variability)		Taxonomic approach (mean population variability)	
	Before	After	Before	After	Before	After
Food web properties						
S (nodes)	130 (59)	120 (36)	71 (59)	50 (36)	71 (59)	49 (36)
Trophic links (L)	482	398	725	389	304	176
Basal level fraction	0.16	0.28	0.17	0.24	0.17	0.27
Intermediate fraction	0.69	0.63	0.70	0.69	0.52	0.41
Top fraction	0.15	0.09	0.13	0.06	0.31	0.33
Trophic level	3.34 ± 0.14***^, a^	2.69 ± 0.11^a^	3.15 ± 0.14^a^	2.91 ± 0.19^a^	2.35 ± 0.09*^, b^	2.01 ± 0.10^b^
Link properties						
Linkage density (L/S)	3.71 ± 0.15^a^	3.32 ± 2.24^a^	10.21 ± 0.72***^, b^	7.94 ± 0.77^b^	4.28 ± 0.36^a^	3.59 ± 0.39^a^
Connectance (2L/S^2^)	0.06	0.06	0.29	0.31	0.12	0.15
Chain properties						
N° chains	37,398***^, a^	18,472	> 10^5^	> 10^5^	1563***^, b^	361
Mean chain length	6.67 ± 0.01	7.12 ± 0.01	–	–	4.05 ± 0.04	2.88 ± 0.07
Omnivory properties						
Degree of omnivory	0.77	0.59	0.82	0.73	0.66	0.61
Degree of intraguild predation	0.10	0.07	0.39	0.41	0.00	0.00
Competition						
N of competitors	13.91 ± 0.47^a^	13.29 ± 0.65^a^	49.97 ± 0.98***^, b^	32.81 ± 0.85^b^	35.39 ± 1.52***^, c^	24.83 ± 1.29^c^
Competition strength (α)	0.35 ± 0.01***^, ab^	0.28 ± 0.09^a^	0.32 ± 0.02^a^	0.36 ± 0.02^b^	0.33 ± 0.01^b^	0.40 ± 0.03^c^
Vulnerability to diversity loss						
Robustness***	0.35	0.39	0.44	0.43	0.17	0.24
Network properties						
Neighbourhood connectivity (NC)	8.39 ± 0.12***^, a^	7.71 ± 0.14^a^	23.36 ± 0.32***^, b^	17.72 ± 0.40^b^	14.07 ± 0.52***^, c^	11.67 ± 0.60^c^
Compartmentalisation	0.03	0.03	0.18	0.20	0.10	0.13
*Core* link properties						
Linkage density (L/S)	1.26 ± 0.12^a^		4.00 ± 0.41^b^		2.11 ± 0.26^c^	
Connectance (2L/S^2^)	0.03		0.20		0.09	

The number of animal species in each period is shown in brackets. Asterisks indicate significant differences between periods (Kruskal–Wallis and associated Mann–Whitney pairwise tests, ANCOVA test for equality of means; * indicates < 0.05, ** < 0.01 and *** < 0.001). Superscript letters (^a^,^b^,^c^) indicate differences between food web approaches. Statistical summary: TL Hc: 60.21; Linkage Density Hc: 93.97 (overall food web) and 176 (food web *core*); Competition α Hc: 48.29; Number of competitors Hc: 309.4; NC Hc: 332.6. The dash indicates that it was not possible to obtain the average chain length due to the high number of chains (> 10^5^) in the web^143^ (Hudson et al., 2013). For the list of species in each web, refer to Tables S1 and S2.

**Table 2:** Proportional contribution of basal and animal-derived food resources to the diet of the benthic community before and after sea-ice breakup for each food web type (ITU, population and taxonomic).

Food source	ITU-based approach (trophic-functional variability)		Population-based approach (intrapopulation variability)		Taxonomic approach (mean population variability)	
	Before	After	Before	After	Before	After
S (nodes)	130 (59)	120 (36)	71 (59)	50 (36)	71 (59)	49 (36)
Sympagic algae	0.21 ± 0.04	0.19 ± 0.05	0.13 ± 0.04	0.13 ± 0.08	0.13 ± 0.04*	0.21 ± 0.06
Seal faeces	–*	0.06 ± 0.04	–	–	0.09 ± 0.04	0.09 ± 0.03
Sediment	0.13 ± 0.02	0.12 ± 0.02	0.16 ± 0.03	0.17 ± 0.03	0.17 ± 0.03	0.21 ± 0.04
Benthic algae	0.10 ± 0.01	0.11 ± 0.02	0.07 ± 0.01	0.06 ± 0.02	0.13 ± 0.06	0.05 ± 0.02
Epiphytes	0.09 ± 0.02	0.11 ± 0.03	0.04 ± 0.01*	0.09 ± 0.02	0.10 ± 0.04	0.09 ± 0.06
Phytoplankton	0.10 ± 0.04	0.09 ± 0.02	0.07 ± 0.02	0.05 ± 0.01	0.11 ± 0.04	0.12 ± 0.02
Animals	0.36 ± 0.03	0.33 ± 0.03	0.52 ± 0.03	0.50 ± 0.04	0.29 ± 0.04*	0.19 ± 0.05

The proportional contribution of each item is reported as the mean ± SE. The number of animal species in each period is shown in brackets. Superscript symbols (*) indicate a significant difference between periods (*p* < 0.05). For the list of species in each web, refer to Tables S1 and S2.

### Diets at different biological scales (community, population, individual)

Isotopic distances between ITUs reflected differences in diet (Mantel test, before: R = 0.03, *p* < 0.001; after: R = 0.43, *p* < 0.001). At the community level, animal matter (whether living or as carrion) was a major contributor to diet, together with sympagic algae and sediments both before and after sea-ice breakup (Table 2). Its importance was highlighted by the population-webs, which took account of individual variability. In both periods the coarse sediment fraction was mainly composed of animal-derived material, which also formed a large percentage of the fine and fine-ultrafine sediment fractions (Tables 3, S3). By contrast, the ultrafine sediment fraction was exclusively composed of primary producers, mainly sympagic algae before sea-ice breakup and a combination of sympagic algae and phytoplankton afterwards. In the fine and fine-ultrafine fractions, benthic algae were the predominant primary producer after sea-ice break-up.

**Table 3:** Contribution (%) of carbon sources to each sediment fraction: coarse (> 1.00 mm), fine (1.00–0.75 mm), fine-ultra-fine (0.75–0.56 mm), ultra-fine (< 0.56 mm), before (a) and after (b) sea-ice breakup.

Source	Sediment fraction			
	Coarse (> 1 mm)	Fine (1–0.75 mm)	Fine-ultra-fine (0.75–0.56 mm)	Ultra-fine (< 0.56 mm)
(a)				
Sympagic algae	0	10.2%	2.9%	100.0%
Benthic algae	0	0	10.1%	0
Epiphytes	9.1%	6.6%	3.0%	0
Phytoplankton	0	0	5.1%	0
Animal-derived matter	90.9%	83.3%	78.8%	0
(b)				
Sympagic algae	0	7.7%	6.6%	76.2%
Benthic algae	0	25.1%	18.4%	0
Epiphytes	8.1%	0	6.9%	0
Phytoplankton	0	0	5.5%	23.8%
Animal-derived matter	91.9%	67.3%	62.5%	0

For the detailed list of species contributing to each sediment fraction, refer to Table S3.

**Figure 5:**
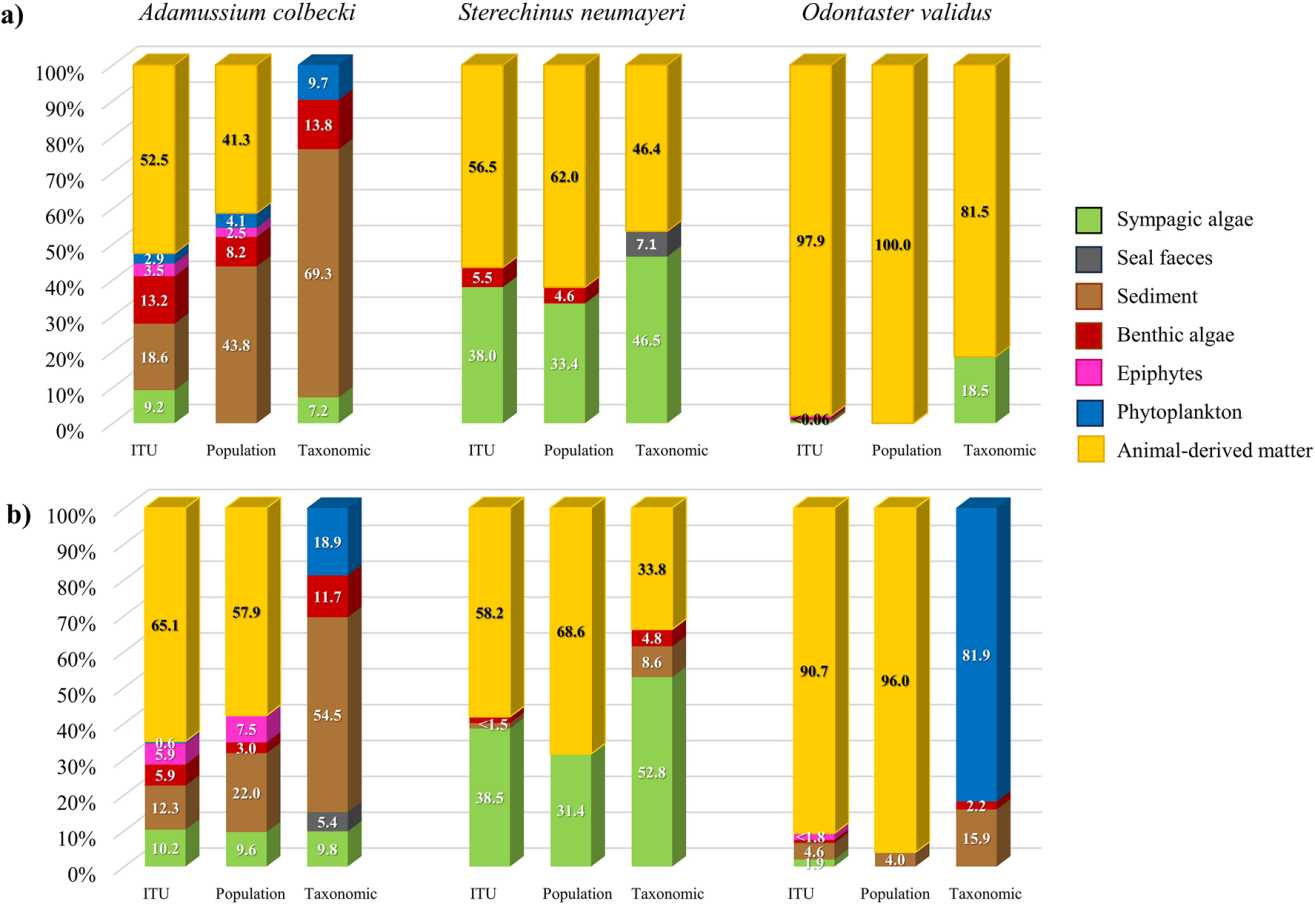
Contribution (%) of basal and animal-derived food resources to the diets of *Adamussium colbecki, Sterechinus neumayeri* and *Odontaster validus* before (**a**) and after (**b**) sea-ice breakup for ITU-webs, population-webs and taxonomic-webs.

Focusing on three of the most abundant macroinvertebrate species in Antarctica (Fig. 5), the scallop *Adamussium colbecki* based its diet largely on the fine and ultra-fine organic sediment fractions. However, for some individuals sympagic algae made up a large proportion of its diet both before and after sea-ice breakup (respectively 9.2 and 10.2% in ITU-webs, 0% and 9.6% in population-webs and 7.20% and 9.8% in taxonomic-webs). The sea-urchin *Sterechinus neumayeri* was found to have a more omnivorous diet, with sympagic algae making up a large proportion both before and after sea-ice breakup (respectively 36.0% and 41.3% in ITU-webs, 33.4% and 31.4% in population-webs and 46.5% and 52.8% in taxonomic-webs). The sea star *Odontaster validus* was a top predator that preyed primarily on *S. neumayeri*, which formed a greater proportion of its diet before than after sea-ice breakup. This trend was highlighted by the ITU-webs and population-webs but not taxonomic-webs (respectively 25.4% and 17.5% in ITU-webs, 28.6% and 9.1% in population-webs, Kruskal–Wallis and Mann–Whitney paired comparison test, Hc: 12.9, *p* always < 0.001; and 20.8% and 25.0% in taxonomic-webs). The contribution of animal matter (whether living or as carrion) was remarkably high in all three species in the ITU-webs and population-webs, but comparatively low or absent in the taxonomic webs, which were based on average isotopic values and literature data (Fig. 5).

### Centrality and compartmentalisation

The three common species (*A. colbecki, S. neumayeri* and *O. validus*) were also central species that played a key role in connecting other species in the food web (i.e. topologically central species; see Table S2, in which other topologically central species are reported). Specifically, *A. colbecki* and the predaceous large sea-star *Diplasterias brucei* were central species in all types of food web, the former both before and after sea-ice breakup and the latter only afterwards.

**Figure 6:**
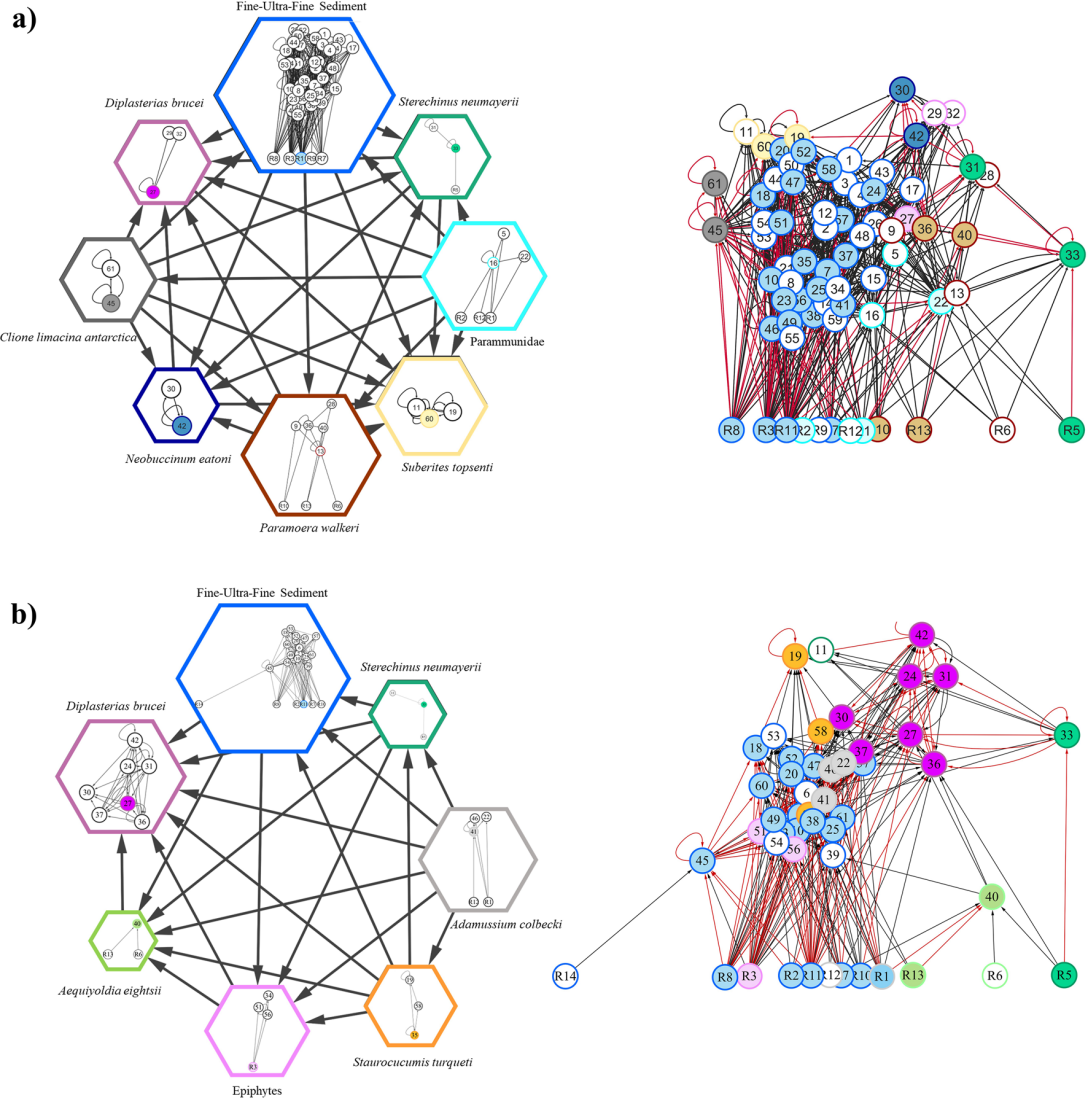
Food web compartments (left) and respective food web structures (right) (**a**) before (November–December 2016) and (**b**) after (January–February 2017) sea-ice breakup (i.e. low and high resource availability respectively) in Tethys Bay. Hexagons represent compartments and arrows are links directed towards the target compartment (left) or consumer (right). The respective sub-network is contained within each compartment. Colours represent compartments (left) and the central nodes in the connections within and between those compartments. The filled nodes and red arrows indicate nodes and links belonging to the population-based web *core*.

Focusing on the central nodes, 8 compartments before and 7 compartments after sea-ice breakup were found in the population-webs, which were the most complex and compartmentalised (Fig. 6). In both periods the compartments were connected by the sea-urchin *S. neumayeri*, the sea-star *D. brucei* and the fine-ultrafine fraction of sediments as a basal resource (Fig. 6).

### Food web core

**Figure 7:**
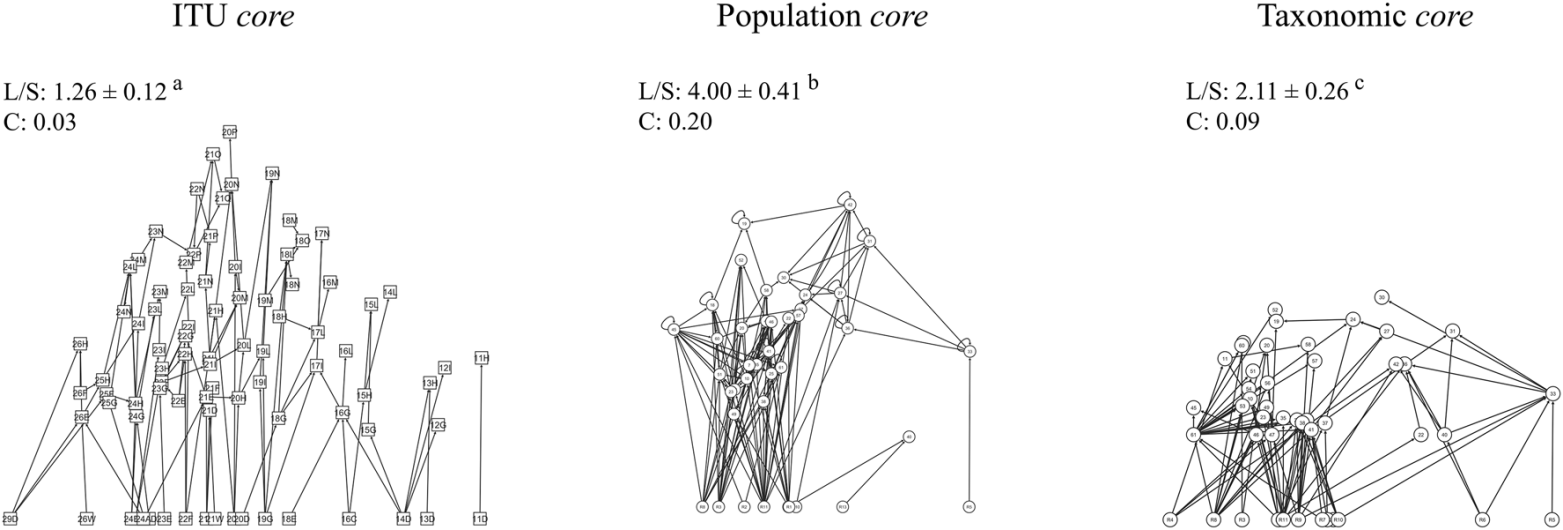
Web *cores* for ITU-webs, population-webs and taxonomic-webs in Tethys Bay. Superscript letters (^a^,^b^,^c^) indicate differences in linkage density between web types (Kruskal–Wallis and Mann–Whitney paired comparison test, Hc: 176, *p* < 0.001).

A web *core* (i.e. an unchanged group of nodes and links including central nodes) was found in each type of food web (Fig. 7). It comprised 86 nodes and 108 links in the ITU-webs, 40 nodes and 160 links in the population-webs and 45 nodes and 97 links in the taxonomic-webs.

The web *core* was significantly simpler in terms of connectance and linkage density than the food webs as a whole (Kruskal–Wallis and Mann–Whitney paired comparison test, Hc: 176, *p* always < 0.001; Fig. 7). Between 30 and 60% of the unchanged links were between basal resources and their consumers. In the ITU-based core web, links to sympagic algae, epiphytes, phytoplankton, benthic algae and fine sediments tended to be preserved. Focusing on the three common species, the sea-urchin *S. neumayeri* preserved links with sympagic algae, which represented its only food source in the population-web *core*, and with its predator *O. validus.* In contrast, the bivalve *A. colbecki* preserved links mainly with benthic algae and fine sediments, although a few individuals preserved links with resources of animal origin.

## Discussion

Understanding the relationship between food web structure and community stability is still one of ecology’s greatest challenges^1–3,25,61–66^. Although the debate is ongoing^67,68^, analysis of random food webs shows that the more complex the community, the less resilient it is^2–4,65,67–70^. However, in nature, interactions between populations within communities are not random^39,54,62,69,71^ and can change with the environmental conditions^6,61,72^. Kondoh^73^ found that short-term variations in trophic links, driven by adaptive consumer food preference, are crucial for the long-term stability of complex communities. This dynamic reorganisation of the food web helps communities to buffer environmental fluctuations.

Antarctic benthic communities are complex but also appear resilient to seasonal disturbances (e.g. iceberg scouring and oscillations in light and temperature^33,74,75^). In the short to medium term, they exhibit the ability to recolonise disturbed areas^18,74–78^ or quickly exploit new and abundant seasonal resources^9,12,17,56,57^. To disentangle the factors underlying this apparent complexity-stability paradox^3,38,39^, in our study the food webs were reconstructed at different biodiversity scales (from individuals to communities). Some crucial points emerged from our results: (1) web structure varies with the availability of basal resources, significant differences being observed between the situation before and after sea-ice breakup, and the responses are seen at all scales; (2) complexity is higher when considering intraspecific variability than webs based on average species’ diets, although the stability can be explained by higher compartmentalisation, i.e. the degree to which food webs are organised into sub-networks^79^; and (3) a web *core*, consisting of the minimal stable structure with nodes and links persisting over time, represents a highly stable food web underlying the highly biodiverse Antarctic communities regardless of resource availability and environmental conditions.

We observed that consumer trophic generalisation decreased with increasing per capita availability of basal resources due to sea-ice breakup. Further, in accordance with *optimal foraging theory*^53,80^, this inverse trend occurred at all scales (individual, population and community)*.* This change was reflected in a reduction in linkage density and the other main food web metrics. Indeed, the foraging choice of individuals has important effects on the entire web structure and stability by determining the level of web complexity, as already predicted by diet breadth models^81^. Connectance, linkage density, direct and indirect interconnection (Neighbourhood Connectivity) and food chain length, all measures of web complexity, as well as robustness to diversity loss, were greater when taking account of individual feeding choices within species (population-webs) than when considering only the average stable isotope signatures for each species (taxonomic-webs; see also^10^). The differences were not due to the number of nodes and thus trophic aggregation (sensu^82,83^).

In contrast, we observed a lower linkage density in the ITU-webs than in the other two web types. This was related to the rather homogeneous distribution of individuals in the isotopic space and reflected a differential use of basal resources. We also observed that after sea-ice breakup intraspecific variability in terms of the number of ITUs per species remained unchanged while the average number of species per ITU was lower. This supports the reduction of interspecific overlap due to diet specialisation when, according to the literature^10,74,84^, the per capita availability of resources increases and the number of species decreases.

The contribution of individual specialisation to foraging diversity, favouring niche differentiation between consumers within a given trophic level, has already been observed^9,85–87^. This is relevant, since despite not being a rare trophic strategy, the specialisation of individuals on different resources in a generalist population (sensu^88^) is rarely considered in food web studies, which do not generally take account of intra-population phenotypic variability^9,89–91^.

Low direct and indirect interconnection in the ITU-webs (NC) was also observed, being even lower after sea-ice breakup, and this may reduce the food web’s vulnerability to the propagation of disturbances^10,92–94^. In accordance with Stewart et al.^95^, the intraspecific specialisation of individuals on distinct food sources can be considered an analogue of compartmentalisation and an emergent property of food webs. Many studies based on empirical models have observed that in nature, the organisation into *‘blocks’* of highly complex communities reduces the instability of the food web^3,4,39,70,96^. The compartmentalisation promoted by intraspecific trophic diversity, combined with the presence of nodes as connectors between compartments observed in this study, could explain the stability of complex Antarctic benthic communities, in which high trophic generalisation, omnivory and intraguild predation are all common^9,14,32,70,79,94,97,98^.

In each type of food web we observed the presence of an invariant subset of nodes and links, or web *core*, which persisted regardless of resource availability. In recent decades, researchers have hypothesised the existence of minimal structures that allow the community to maximise energy transfer at ‘minimal cost’ through key pathways within the food web^99–101^. Food web complexity, in terms of linkage density and connectance, was lower in the web *core* than in the food web as a whole. This suggests that the web *core* actually represents the minimal structure, highly stable over time, of a complex community that opportunistically adds new links based on resource availability and consumers’ adaptive foraging behaviours^73,99,102,103^. In these sub-webs, the trophic variability of the central species is maintained. Together with compartmentalisation, the presence of a trophic-functional web *core* may provide the key to understanding the extraordinary persistence of complex Antarctic communities.

Sympagic algae, sediment, and animal matter were the most important carbon sources in the food webs. This is consistent with several studies showing that sympagic algae and organic matter storage in the sediment are among the resources that most support Antarctic benthic communities in both medium-shallow and deep waters^9,17,104,105^. The former is an energy-rich resource whose availability is limited to a short period of the year, i.e. the end of spring and the beginning of summer^23,55,56,106^. During this period, sea-ice thinning due to increased temperature increases light radiation, favouring the growth of sympagic algae that are released when the sea ice breaks up and melts^21,23,55,56^. In contrast, sediment represents an important food bank mostly during the unproductive season, powered by seasonal depositions of particulate organic matter from various sources^55,107,108^. Our study shows that the composition of organic sediment in Tethys Bay varied from almost exclusively animal-derived matter in the coarse fraction, both before and after sea-ice breakup, to solely sympagic algae before sea-ice breakup and both sympagic algae and phytoplankton afterwards in the ultra-fine fraction. After sea-ice breakup, in the intermediate fractions of the sediment, i.e. fine and fine-ultrafine, the previously large contribution of animal-derived matter decreased, while that of benthic algae increased. The fine-ultrafine sediment fraction was an important connector of food web compartments in both periods, confirming its important role as a persistent food source for benthic organisms^108^.

Variation in the contribution of carbon sources to diets was observed in the two omnivorous key species, the scallop *A. colbecki* and the sea-urchin *S. neumayeri*. Sediments and animal matter were the main carbon sources for the scallop, whose consumption of sympagic algae was higher after sea-ice breakup, while the sea-urchin was able to consume sympagic algae both before and after. By clapping, the bivalve *A. colbecki* is able to resuspend and filter the organic matter deposited in the sediment^109,110^. It can thus exploit the sympagic algae in the sediments in periods of low availability and those released by melting ice in periods of high availability. The sea star *O. validus* exhibited highly predatory behaviour. Its diet relied mostly on the sympagic chain, consuming mainly the sea urchin in both periods. These three common central species are also important to the web core, playing a key role in carbon processing and transfer, as well as in benthic-pelagic-sympagic coupling over time^97,98,109^.

The substantial contribution of animal matter to the diets of consumers in the food webs, including scallops, was revealed by the ITU-based analyses. Conventional diet reconstruction methods, based on species’ average trophic preferences, require a priori awareness of possible resources to include in the models^63^. Pre-assigning a restricted set of possible resources based on limited or inadequate scientific information on the diet of target species can represent a further impediment to the accurate reconstruction of food webs^9,27,63^. The ITU-based approach helps overcome this limitation, allowing a more accurate estimate of consumers’ diets and thus yielding useful information for effective ecosystem management measures in both the short and long term.

In this kind of study, tissue turnover may constitute a limitation on the interpretation of mixing model results^104^. However, determining turnover rates is particularly complicated in food webs with many species, since it is costly and time-consuming, and the relevance of the estimates to other species is not clear^111^. It is particularly challenging in Antarctica due to logistics. Although we know of no experiments determining isotopic fractionation in Antarctic benthic invertebrate organisms, some studies of their metabolism support the hypothesis that fractionation increases in periods of greater food availability^112,113^. Indeed the metabolism and growth rates of Antarctic marine species are significantly affected by seasonal cycles and the availability of food sources^113–117^. While they display slow metabolism for most of the year^76,112,113^, in the most productive period, during sea-ice breakup, metabolic and growth rates increase^112,113,115,118,119^. Increasing metabolic rate is associated with increasing tissue turnover and changes in isotopic ratios^46,50,120^. Kaufman et al.^120^ reported a tissue turnover rate of about 3 weeks in an Arctic amphipod in high-productivity periods, and Weems et al.^121^ observed similar results in two Arctic bivalves. Consistent with these observations, the tissues collected from Antarctic invertebrates might indicate recent dietary shifts. Calizza et al.^17^ showed the consumption of sympagic algae even before sea-ice breakup by isotopic analysis of muscles, and consumption of sympagic algae and phytoplankton only 3 days after complete sea-ice disappearance by gut content analysis.

Laboratory measurements of Antarctic invertebrate tissue turnover rates would improve the accuracy of attempts to quantify temporal changes in their diets on an isotopic basis. The incorporation of additional biochemical tracers, such as fatty acids^122^, and isotopic analysis of specific amino acids^123^ could further enhance our ability to discriminate between the different sources of carbon that fuel complex food webs such as those found in Antarctica.

In conclusion, the persistence of high levels of biodiversity can be explained by the ability of organisms to adapt their diet to different conditions of food availability^12,27,28^, seasonally rewiring the food webs. This ability varies even within the same population of a given species^27,28,30^. Neglecting intrapopulation variability in food choice could result in underestimation of species’ diets and thus oversimplification of food web structure and complexity, leading to possible misinterpretation of the mechanisms underlying community stability^9,27,34,63,89,124^.

Understanding the actual structure and functioning of food webs is critical to making predictions about the increasingly widespread impacts of climate and other environmental change on communities and ecosystems^6,11,69,104,125^. However, the reconstruction of food webs is still affected by several limitations, mainly due to the difficulty of analysing communities with high trophic and taxonomic diversity^9,13,27,29,30,35,126^. In addition, taxonomic resolution is not always homogeneous, and generalisation of observations is often impeded by the different approaches and non-comparable datasets adopted across sites and periods^127^. By grouping individuals with similar tropho-functional traits, the ITU-based approach provides an effective method for describing in detail and comparing food web architecture even when communities are extremely complex and biodiverse in variable environments such as Antarctica.

The detailed description of populations’ diets based on the diet of each individual made it possible to identify the role of each population in the food web and the set of resources actually assimilated by individuals. This enabled us to identify not just a core web persisting independently of resource availability and environmental conditions, but also ‘*community hubs’.* These are sets of key species with high trophic plasticity, which play a key role in maintaining web structure and therefore ecosystem functions^128,129^ and energy flows in Antarctic benthic ecosystems^9,17,98,104^.

Comparing food web structure before and after sea-ice breakup has proven to be a powerful tool for studying the mechanisms underlying the architecture of biodiversity maintained by predictable energy supply. This yielded knowledge needed to predict community response to environmental change. For the purposes of managing and conserving complex communities and key species threatened by environmental change, it is therefore appropriate to first consider and describe in detail the trophic-functional traits and diet of community members. This will provide an accurate representation of the architecture of biodiversity and predict future scenarios.

Climate projections suggest a significant reduction in sea ice cover across Antarctica, with the exception of the Ross Sea^130,131^. Changes in sea ice cover, including alterations in seasonal dynamics, ice thickness, annual ice loss, and variations in sunlight diffusion^15,84^ can alter availability of energy-efficient resources (e.g., sympagic algae), which are crucial for the survival and reproduction of benthic organisms^21,55,56^.

Our findings suggest that taxa in these communities, typically adapted to an extremely stable environment^18,20,74,77^, could adapt their dietary habits further by increasing trophic generalisation during prolonged periods of limited resources. The results are consistent with other studies conducted both in the same area and elsewhere, at a range of depths^9,14,17,104,132^. These studies indicate that variations in sea ice coverage could affect the structure of the Antarctic benthic food web on various spatio-temporal scales. In addition to reduced ice algae production, the reduction of seasonal sea ice coverage could raise the frequency of catastrophic events such as iceberg drift, especially in shallow coastal waters^33,74,75,133^. This would increase the availability of animal carcasses, either fresh or stored in sediments, favouring necrophagy among Antarctic organisms^77,133^. It has also been demonstrated that conditions of extreme sea ice reduction, or absence, result in decreased Antarctic benthic biodiversity^15,84^. Such conditions are already evident in some areas of the Antarctic Peninsula and sub-Antarctic regions, which exhibit growing vulnerability to the local loss of the most connected species^65,94^. In contrast, multiyear sea ice is common in McMurdo Sound and New Harbor^134^. Norkko et al.^14^ found that compared to fresh algal rich areas near ice-free waters, in conditions of high sea-ice persistence and thickness, taxa shift towards omnivory, primarily based on sediments and detritus. Michel et al.^104^ showed that the multiyear absence of sea-ice breakup but low sea-ice thickness (< 200 cm), favours algal growth. Under these conditions, benthic consumers base their diet mostly on the sympagic chain, reducing omnivory and lowering trophic positions. These changes in the food web structure at the benthic level could have effects on higher trophic levels in the Antarctic ecosystem^20,135^.

## Materials and methods

### Study area and sampling

The study area is Tethys Bay (74° 41′ 8″ S, 164° 04′ 8″ E), a 3 km-long inlet of Terra Nova Bay in the Ross Sea MPA (Antarctica) characterised by high biodiversity^9,136^. It hosts several species of gastropods, echinoderms (asteroids, ophiuroids, holothuroids, echinoids and crinoids), bivalves, pycnogonids, sponges, polychaetes and many other macroinvertebrates^9,136,137^.

The marked seasonality in the dynamics of sea-ice coverage, breakup and melting in the bay regulates the availability of food for benthic communities^10^. Sampling was carried out both in the period of maximum ice thickness (from mid-November to mid-December) and at least 31 days after the start of sea-ice breakup, which causes massive release of sympagic algae. Sea-ice breakup began on 18 December 2016, leaving the sea completely ice-free by around 10 January 2017. The sampling periods reflect the timing of tissue turnover in polar benthic marine invertebrates during the most productive season^120^, before the shift to a phytoplankton-based diet, typical of ice-free conditions, can take place^17,21,57,97,109,110^.

Samples were collected by SCUBA divers at depths of 10–30 m. Macroinvertebrates and basal resources including micro and macroalgae (*Iridaea cordata* and *Phyllophora antarctica*), together with associated epiphytes, Weddell seal faeces and sediments were sampled. Sympagic algae were collected by hand by scraping ice cores. Sediments were fractionated as Coarse (> 1.00 mm), Fine (1.00–0.75 mm), Fine-Ultra-Fine (0.75–0.56 mm) and Ultra-Fine (< 0.56 mm). A total of 1732 specimens belonging to 61 different animal species were sampled and processed. Approximately half were common before and after sea-ice breakup. For the isotopic analysis, tissues of large invertebrates were used, while small individuals were analysed whole or pooled to reach sufficient biomass for the analysis. Samples were then freeze-dried, ground into a fine powder and analysed. Where necessary, samples were pre-acidified using drop-by-drop 1 M HCl^138^ to remove inorganic carbon, which can interfere with the δ^13^C signature, while δ^15^N was measured in non-acidified powders^9,17,46^. Samples were analysed in two replicates with a mass spectrometer (IsoPrime100, Isoprime Ltd., Cheadle Hulme, United Kingdom) coupled with an elemental analyser (Elementar Vario Micro-Cube, Elementar Analysensysteme GmbH, Germany). The results (δ^13^C and δ^15^N) are expressed in delta units per thousand (‰) relative to international standards and indicate the presence of heavier isotopes. Further details on the study area and sampling and lab activities can be found in Sporta Caputi et al.^10^.

### Food web reconstruction

Isotopic Trophic Units (ITUs) based on the isotopic data of individual organisms were used to reconstruct the communities’ trophic-functional web structure. The Isotopic Trophic Units were defined as single individuals or groups of individuals having similar carbon and nitrogen isotopic signatures^9^, i.e. occupying the same position within the isotopic space, considered as trophospecies (sensu^139^). ITUs were identified by subdividing the bi-dimensional isotopic space into squares corresponding to 1‰ on the δ^15^N and δ^13^C axes, starting from the lowest δ^13^C value in the dataset and a δ^15^N value of zero (Fig. 1). Each ITU was labelled with a number and a letter corresponding to its isotopic values on the δ^13^C and δ^15^N axes respectively.

The 1‰ intervals defining the ITUs (corresponding to 1‰ × 1‰ squares in the biplot) represent the minimum units for comparing communities with different isotopic signatures in time and space^9^. These dimensions also allow us to reconstruct food webs with nodes occupying equal portions of the community isotopic space, reducing the potentially large and variable numbers of individuals and species included in each ITU. This, in turn, allows for better and more accurate extrapolation of trophic links between species. Since the maximum isotopic carbon difference between a consumer and its resource is 1‰ among aquatic animals^50^, differences greater than 1‰ on the δ^13^C axis may be indicative of dietary differences between individuals. To confirm this, as in Rossi et al.^9^, the correlation between the similarity in trophic links and the isotopic distance between ITUs was verified by means of a Mantel test. While the isotopic discrimination factor for nitrogen is generally broader (~ 2.3‰ for invertebrates^9,50^), the 1‰ resolution on the δ^15^N axis allows for the categorisation of individuals belonging to omnivorous populations into different trophic units. This preserves information on the vertical distribution of individuals along the food chains and the complexity of the food web.

The diet of each ITU was estimated by means of Bayesian Mixing Models (SIMMr package, R-software ver. 4.1.1^140,141^), considering Trophic Enrichment Factors of 0.4 ± 0.2‰ for δ^13^C and 2.3 ± 0.4‰ for δ^15^N^9,10,17^. The model required three inputs: (1) the isotopic signatures of the ITU consumer, (2) the isotopic signatures (mean and standard deviation) of the ITU consumer’s food sources and (3) the ITU consumer’s Trophic Enrichment Factor (TEF). The uncertainties considered in the TEF values make it possible to take into account any variability due to differing assimilation of the various food source items by each consumer^30,44,50,142^. The model output provides probability distributions of the proportional contribution of each food source to the diet of each consumer expressed as mean ± standard deviation and respective upper and lower limits of credibility ranges (CI 50%, 75%, 95%).

In order to obtain the actual resource pool for each consumer, several steps based on the mixing model outputs were performed in accordance with Rossi et al.^9^. The contribution of the various organic carbon sources to each sediment fraction was estimated both about 4 weeks before and 4 weeks after sea-ice breakup, without incorporating trophic enrichment into the model.

At population level, in both sea-ice periods, the diet of each species was determined by analysing the diets of the ITUs including specimens of that species^9,30^. Similar to Rossi et al.^9^, we assumed that all food sources consumed by specimens within ITUs were equally likely to be included in the overall diet of the consumer population. Consequently, the overall contribution of a specific food source to each consumer's diet will be important at the individual level (> 5%) but less (< 5%) at the population level^30^. To obtain detailed information on the diet of each population, and therefore on the structure of the food web, we considered only food sources with an overall contribution to a population’s diet of > 2%. This was to exclude resources whose standard deviation could result in values close to zero. These resources were basal and animal items that (1) were generally poorly explored by population individuals, or (2) were consumed by only one or a few individuals, contributing less on average to the diet of the population as a whole than other items.

Both ITU-webs and population-webs were compared with the ones published in Sporta Caputi et al.^10^. In the latter (taxonomic-webs) the potential food sources for each species were selected based on the literature^14,17,97,104,110,124^ and/or their position in the bi-dimensional isotopic space.

The web *core* was considered as the minimum existing structure, considering nodes and links common to both pre- and post-sea-ice breakup conditions.

### Food web metrics and data analysis

After assigning trophic links, web metrics were obtained using Cytoscape 3.9.1 and the Cheddar and NetworkExtinction packages in R-software ver. 4.1.1^143,144^.

The nodes (S) in the webs were either ITUs or species, while the links (L) were the feeding links from resources to consumers. The linkage density (L/S) is the average number of feeding links per node while connectance (C) is:

$${\text{C }} = {\text{ 2L}}/{\text{S}}^{{2}} .$$

Potential competition for resources was measured both as the average number of nodes sharing at least one resource and as resource use overlap (α) in accordance with Levins’ (1968) equation:

$$\alpha_{i,j} = \frac{{\mathop \sum \nolimits_{k = 1}^{N} p_{ik} *p_{jk} }}{{\mathop \sum \nolimits_{k = 1}^{N} \left( {p_{ik} } \right)^{2} }}$$

where *i* and *j* form a pair of overlapping nodes, and *p*_*ik*_ and *p*_*jk*_ are the proportional contributions of resource* k* to the diets of *i* and *j* respectively. For each node, the Trophic Level (TL) was also calculated in accordance with the formula:

$${\text{TL}}_{i} = { }1 + { }\mathop \sum \limits_{k = 1}^{N} {\text{TL}}_{k} *p_{ik}$$

where* i* is the target consumer node, *k* the resource item and *p*_*ik*_ the proportional contribution of resource* k* to the diet of *i*^145^. The TL of basal resources was 1.

Neighbourhood Connectivity (NC), Betweenness Centrality (BC) and web robustness to node loss were also estimated. NC measures the degree of interconnection (direct and indirect) between nodes in the food web, while BC is a topological measure of the centrality of a node in a food web^10,146,147^. The top ten nodes (ITUs or species) in terms of their role in connecting other nodes or compartments in the food web were identified with reference to their BC values. The ModuLand plug-in for Cytoscape^148^ was used to identify the compartments of the food web and the role of nodes in connecting them. Web robustness was quantified by simulating primary extinctions (sensu^71^) from the most to the least connected node within the food web^9,10^. This represents the worst possible extinction scenario, giving us useful information on the maximum vulnerability of communities to diversity loss. Secondary extinction occurs when a consumer node loses all its resource items, and the robustness index was expressed as the proportion of primary losses leading to the primary or secondary loss of 50% of all community nodes^71^.

The metrics of the food webs before and after sea-ice breakup, hence under conditions of low and high resource availability, and of the three types of food web (ITU-webs, population-webs and taxonomic-webs) were compared. Due to computational limits, which do not allow chains of > 10^5^ to be accurately estimated^143^, quantifying the exact mean chain length in the population-web was not possible. Comparisons were also made at population level by focusing on key species in terms of their centrality in the food web (sensu Martín González et al.^147^).

Before performing statistical comparisons, the Shapiro–Wilk normality test was applied to each dataset to verify the Gaussian distribution and then use the most appropriate statistical tests. A Mann–Whitney test was performed to determine the difference in the mean number of species per ITU before and after sea-ice breakup. A Mantel test was then applied in order to determine whether the isotopic signatures of ITUs were predictive of the feeding links they established in the food web^9,10,149^. The correlation test was performed between the matrices of isotopic Euclidean distances and the Bray–Curtis diet similarity^9,10,149^.

Kruskal–Wallis and Mann–Whitney pairwise tests were used to compare linkage density, Neighbourhood Connectivity, trophic level and the number of competitors, as well as resource consumption before and after sea-ice breakup in the three food web types. Finally, ANCOVA with primary extinction as a covariate factor was used to test the difference between web types in terms of web robustness to node loss.

Unless otherwise specified, the results are reported as mean ± standard error (SE). In order to exclude differences arising from the scale of trophic aggregation (i.e. ITU-webs, population-webs and taxonomic-webs), the relationships between (1) the number of nodes in the webs and the proportion of basal, intermediate and top nodes and (2) the number of nodes and the web metrics were tested in accordance with Martinez and Lawton^83^.

### Supplementary Information

Supplementary Tables.

## Data Availability

The datasets used and/or analysed during the current study are available from the corresponding author on reasonable request.
