# Supplementary material for: Individual diet variability shapes the architecture of Antarctic benthic food webs
Source: Sci Rep. 2024 May 29;14:12333. doi: 10.1038/s41598-024-62644-5 (PMC11137039; doi:10.1038/s41598-024-62644-5)
Supplement: Supplementary file 1 — Supplementary Tables. [file 41598_2024_62644_MOESM1_ESM.docx]

**The role of trophic phenotypes in shaping the architecture of Antarctic benthic food webs: a study based on stable isotope analysis**

Sporta Caputi S.^1,2^, Kabala J. P. ^1^, Rossi L. ^2,^*, Careddu G. ^1,2^, Calizza E. ^1,2^, Ventura M. ^1^, Costantini M.L. ^1,2^

^1^Department of Environmental Biology, Sapienza University of Rome, via dei Sardi 70, 00185 Rome, Italy

^2^CoNISMa, National Inter-University Consortium for Marine Sciences, piazzale Flaminio 9, 00196 Rome, Italy

*Corresponding Loreto Rossi: [loreto.rossi@uniroma1.it](mailto:loreto.rossi@uniroma1.it)

***Supplementary material***

**Table S1** List of taxa/basal resources present in each ITU (Isotopic Trophic Unit) before a) and after b) sea-ice breakup.

| ITU-CODE | TAXON |
| --- | --- |
| 8C | Filamentous algae (ice core) |
| 10G | *Sterechinus neumayeri* |
| 11D | Filamentous algae (ice core) |
| 11G | *Sterechinus neumayeri* |
| 11H | *Sterechinus neumayeri* |
| 12B | Filamentous algae (ice core) |
| 12G | *Sterechinus neumayeri* |
| 12H | *Sterechinus neumayeri* |
| 12I | *Ophionotus victoriae* |
| 12I | *Sterechinus neumayeri* |
| 13C | Filamentous algae (ice core) |
| 13D | Filamentous algae (ice core) |
| 13G | *Sterechinus neumayeri* |
| 13H | *Sterechinus neumayeri* |
| 13I | *Ophionotus victoriae* |
| 14A | Filamentous algae (ice core) |
| 14B | Filamentous algae (ice core) |
| 14D | Filamentous algae (ice core) |
| 14G | *Camptoplites* sp. |
| 14G | *Sterechinus neumayeri* |
| 14I | *Diplasterias brucei* |
| 14L | *Odontaster validus* |
| 14L | *Ophionotus victoriae* |
| 14L | *Sterechinus neumayeri* |
| 15A | Filamentous algae (ice core) |
| 15G | *Paramoera walkeri* |
| 15G | *Sterechinus neumayeri* |
| 15H | *Sterechinus neumayeri* |
| 15L | *Odontaster validus* |
| 16A | Filamentous algae (ice core) |
| 16B | Filamentous algae (ice core) |
| 16C | Filamentous algae (interface) |
|  |  |
|  |  |
| 16F | *Camptoplites* sp. |
| 16G | *Paramoera walkeri* |
| 16G | *Sterechinus neumayeri* |
| 16H | *Sterechinus neumayeri* |
| 16I | *Diplasterias brucei* |
| 16I | *Ophionotus victoriae* |
| 16L | *Diplasteria brucei* (adults) |
| 16L | *Odontaster validus* |
| 16M | *Odontaster validus* |
| 17A | Diatoms (ice core) |
| 17F | *Paramoera walkeri* |
| 17G | *Ophionotus victoriae* |
| 17G | *Paramoera walkeri* |
| 17H | *Aequiyoldia eightsii* |
| 17H | *Diplasterias brucei* |
| 17H | *Ophionotus victoriae* |
| 17I | *Diplasteria brucei* (adults) |
| 17I | *Ophionotus victoriae* |
| 17I | *Ophioplinthus gelida* |
| 17L | *Diplasterias brucei* |
| 17L | *Odontaster validus* |
| 17N | *Neobuccinum eatoni* |
| 17O | *Odontaster* sp. |
| 17Q | *Neobuccinum eatoni* |
| 18C | Diatoms (interface) |
| 18E | Benthic diatoms |
| 18E | Diatoms (ice core) |
| 18F | *Camptoplites* sp. |
| 18F | *Cucumaria* sp. |
| 18F | *Paramoera walkeri* |
| 18G | *Diplasterias brucei* |
| 18H | *Diplasterias brucei* |
|  |  |
|  |  |
| 18I | *Diplasterias brucei* |
| 18I | *Neobuccinum eatoni* |
| 18I | *Nototanais dimorphus* |
| 18I | *Ophionotus victoriae* |
| 18L | *Diplasterias brucei* |
| 18L | *Odontaster validus* |
| 18L | *Urticinopsis antarctica* |
| 18M | *Neobuccinum eatoni* |
| 18M | *Urticinopsis antarctica* |
| 18N | *Acodontaster hodgsoni* |
| 18N | *Neobuccinum eatoni* |
| 18N | *Perknaster fucus antarcticus* |
| 18O | *Odontaster* sp. |
| 19D | Paramunnidae |
| 19E | Paramunnidae |
| 19F | *Orchomenella* sp. |
| 19G | *Camptoplites* sp. |
| 19G | Fine-Ultra-Fine Sediment |
| 19G | *Paramoera walkeri* |
| 19G | *Psamathe fauveli* |
| 19H | *Alcyonium antarcticum* |
| 19H | *Diplasterias brucei* |
| 19H | *Flabelligera mundata* |
| 19H | *Ophionotus victoriae* |
| 19I | *Diplasterias brucei* |
| 19I | *Munna* sp. |
| 19I | *Ophionotus victoriae* |
| 19I | *Parbolasia corrugatus* |
| 19L | Hesionidae |
| 19L | *Neobuccinum eatoni* |
| 19L | *Odontaster* sp. |
| 19L | *Ophionotus victoriae* |
| 19L | Polynoidae |
| 19L | *Urticinopsis antarctica* |
| 19M | *Diplasterias brucei* |
| 19M | *Neobuccinum eatoni* |
| 19M | *Odontaster validus* |
| 19N | *Neobuccinum eatoni* |
| 19O | *Ammothea* sp. |
| 19P | *Odontaster* sp. |
| 19Q | *Odontaster* sp. |
| 19W | Diatoms (ice core) |
| 20D | *Iridaea cordata* |
| 20D | Paramunnidae |
| 20E | Paramunnidae |
| 20F | *Camptoplites* sp. |
| 20F | Fine Sediment |
| 20G | *Camptoplites* sp. |
| 20G | *Ophioplinthus gelida* |
| 20H | *Munna* sp. |
| 20H | *Ophionotus victoriae* |
| 20H | *Ophioplinthus gelida* |
| 20H | Serpulidae |
| 20H | Terebellidae |
| 20I | *Barrukia cristata* |
| 20I | *Clavularia frankliniana* |
| 20I | *Diplasterias brucei* |
| 20I | *Ophionotus victoriae* |
| 20I | *Parbolasia corrugatus* |
| 20L | Hesionidae |
| 20L | *Nototanais dimorphus* |
| 20L | *Odontaster validus* |
| 20L | *Ophionotus victoriae* |
| 20L | Polynoidae |
| 20L | *Trophonella longstaffi* |
| 20L | *Urticinopsis antarctica* |
| 20M | Hesionidae |
| 20M | *Leitoscoloplos kerguelensis* |
| 20M | *Neobuccinum eatoni* |
| 20M | *Urticinopsis antarctica* |
| 20N | *Leitoscoloplos kerguelensis* |
| 20O | *Odontaster* sp. |
| 20P | *Ammothea* sp. |
| 21C | *Adamussium colbecki* |
| 21C | Benthic diatoms |
| 21D | *Adamussium colbecki* |
| 21E | *Adamussium colbecki* |
| 21E | *Iridaea cordata* |
| 21E | Paramunnidae |
| 21F | *Adamussium colbecki* |
| 21F | Fine Sediment |
| 21G | *Adamussium colbecki* |
| 21G | *Alcyonium antarcticum* |
| 21G | *Homaxinella balfourensis* |
| 21G | *Laternula elliptica* |
| 21G | *Nymphon australe* |
| 21H | *Adamussium colbecki* |
| 21H | *Alcyonium antarcticum* |
| 21H | Ascidiacea |
| 21H | *Munna* sp. |
| 21H | *Neobuccinum eatoni* |
| 21H | *Ophionotus victoriae* |
| 21H | *Ophioplinthus gelida* |
| 21H | *Parbolasia corrugatus* |
| 21H | Terebellidae |
| 21I | *Barrukia cristata* |
| 21I | Hesionidae |
| 21I | *Leitoscoloplos kerguelensis* |
| 21I | *Ophioplinthus gelida* |
| 21I | *Polymastia invaginata* |
| 21I | Polynoidae |
| 21L | *Barrukia cristata* |
| 21L | Hesionidae |
| 21L | *Odontaster validus* |
| 21L | Polynoidae |
| 21L | *Sphaerotylus antarcticus* |
| 21N | *Ammothea* sp. |
| 21N | *Sphaerotylus antarcticus* |
| 21O | *Ammothea* sp. |
| 21O | *Odontaster* sp. |
| 21P | *Ammothea* sp. |
| 21Q | *Odontaster* sp. |
| 21W | Diatoms (ice core) |
| 21W | Ultra-Fine Sediment |
| 22E | *Adamussium colbecki* |
| 22E | Fine-Ultra-Fine Sediment |
| 22E | *Iridaea cordata* |
| 22E | *Laternula elliptica* |
| 22E | *Limacina helicina* |
| 22F | *Adamussium colbecki* |
| 22F | Arcturidae |
| 22F | Ascidiacea |
| 22F | *Cucumaria* sp. |
| 22F | Epiphytes |
| 22F | Fine-Ultra-Fine Sediment |
| 22F | *Homaxinella balfourensis* |
| 22G | *Adamussium colbecki* |
| 22G | *Alcyonium antarcticum* |
| 22G | *Barrukia cristata* |
| 22G | *Homaxinella balfourensis* |
| 22G | *Nymphon australe* |
| 22G | *Ophioplinthus gelida* |
| 22G | Serpulidae |
| 22G | Terebellidae |
| 22H | *Adamussium colbecki* |
| 22H | Coarse Sediment |
| 22H | *Diplasterias brucei* |
| 22H | *Staurocucumis turqueti* |
| 22I | *Achelia* sp. |
| 22I | *Barrukia cristata* |
| 22I | *Ophioplinthus gelida* |
| 22I | *Orchomenella* sp. |
| 22I | *Staurocucumis turqueti* |
| 22L | *Orchomenella* sp. |
| 22L | *Parbolasia corrugatus* |
| 22L | *Sphaerotylus antarcticus* |
| 22M | Nematoda |
| 22M | *Sphaerotylus antarcticus* |
| 22N | *Alcyonium antarcticum* |
| 22N | Hesionidae |
| 22N | Nematoda |
| 22P | *Ammothea* sp. |
| 23A | Diatoms (interface) |
| 23D | Fine-Ultra-Fine Sediment |
| 23D | *Limacina helicina* |
| 23E | Fine-Ultra-Fine Sediment |
| 23E | *Limacina helicina* |
| 23E | Phytoplankton |
| 23F | Benthic diatoms |
| 23F | *Dendrilla antarctica* |
| 23F | Fine-Ultra-Fine Sediment |
| 23F | *Limacina helicina* |
| 23G | *Alcyonium antarcticum* |
| 23G | Ascidiacea |
| 23G | *Clione limacina antarctica* |
| 23G | *Dendrilla antarctica* |
| 23G | *Kirkpatrickia variolosa* |
| 23G | *Mycale* sp. |
| 23G | *Nymphon australe* |
| 23G | *Staurocucumis turqueti* |
| 23G | Terebellidae |
| 23H | *Alcyonium antarcticum* |
| 23H | Ascidiacea |
| 23H | Epiphytes |
| 23H | *Haliclona* sp. |
| 23H | *Mycale* sp. |
| 23H | *Ophionotus victoriae* |
| 23H | Spionidae |
| 23H | *Staurocucumis turqueti* |
| 23H | Terebellidae |
| 23H | *Urticinopsis antarctica* |
| 23I | *Achelia* sp. |
| 23I | *Haliclona* sp. |
| 23I | *Hemigellius fimbriatus* |
| 23I | Hesionidae |
| 23I | *Mycale* sp. |
| 23I | Nematoda |
| 23I | *Orchomenella* sp. |
| 23I | Serpulidae |
| 23I | *Urticinopsis antarctica* |
| 23L | *Achelia* sp. |
| 23L | *Haliclona dancoi* |
| 23L | *Haliclona* sp. |
| 23L | *Hemigellius* sp. |
| 23L | Nematoda |
| 23L | *Nymphon australe* |
| 23L | *Tritoniella belli* |
| 23M | *Ammothea* sp. |
| 23M | *Colossendeis* sp. |
| 23M | *Haliclona dancoi* |
| 23M | *Haliclona* sp. |
| 23M | *Haliclona tenella* |
| 23M | *Leitoscoloplos kerguelensis* |
| 23M | Nematoda |
| 23M | *Nymphon australe* |
| 23M | *Tritoniella belli* |
| 23N | *Achelia* sp. |
| 23N | *Suberites topsenti* |
| 24A | Diatoms (interface) |
| 24C | Fine-Ultra-Fine Sediment |
| 24E | Epiphytes |
| 24E | Fine-Ultra-Fine Sediment |
| 24E | Phytoplankton |
| 24F | *Cucumaria* sp. |
| 24F | Epiphytes |
| 24F | *Haliclona* sp. |
| 24F | *Staurocucumis turqueti* |
| 24G | Epiphytes |
| 24G | *Hemigellius fimbriatus* |
| 24G | *Hemigellius* sp. |
| 24G | Terebellidae |
| 24H | Ascidiacea |
| 24H | *Haliclona* sp. |
| 24H | Terebellidae |
| 24I | *Achelia* sp. |
| 24I | *Hemigellius* sp. |
| 24L | *Achelia* sp. |
| 24L | *Ammothea* sp. |
| 24L | *Nymphon australe* |
| 24L | *Suberites topsenti* |
| 24M | *Achelia* sp. |
| 24M | Amphipoda *(alia)* |
| 24M | *Nymphon australe* |
| 24M | *Suberites topsenti* |
| 24N | Amphipoda *(alia)* |
| 25C | *Limacina helicina* |
| 25D | *Suberites topsenti* |
| 25F | *Clione limacina antarctica* |
| 25F | Epiphytes |
| 25F | Terebellidae |
| 25G | *Clione limacina antarctica* |
| 25G | *Diplasterias brucei* |
| 25G | Epiphytes |
| 25G | *Ophionotus victoriae* |
| 25G | Terebellidae |
| 25H | Terebellidae |
| 25H | Zooplankton |
| 25I | Hesionidae |
| 25L | *Achelia* sp. |
| 25L | *Ammothea* sp. |
| 25L | Amphipoda *(alia)* |
| 25P | Seal Faeces |
| 25Q | Seal Faeces |
| 26E | *Clione limacina antarctica* |
| 26F | Ascidiacea |
| 26F | *Clione limacina antarctica* |
| 26F | Zooplankton |
| 26G | Terebellidae |
| 26H | *Alcyonium antarcticum* |
| 26H | *Ophioplinthus gelida* |
| 26Q | Seal Faeces |
| 26W | Diatoms (interface) |
| 27E | *Clione limacina antarctica* |
| 27P | Seal Faeces |
| 28O | Seal Faeces |
| 28Q | Seal Faeces |
| 29D | Phytoplankton |
| 31A | *Phyllophora antartica* |
| 35B | *Phyllophora antartica* |
| 35C | *Phyllophora antartica* |
| 36A | *Phyllophora antartica* |
| 36B | *Phyllophora antartica* |
| 36C | *Phyllophora antartica* |
| 37A | *Phyllophora antartica* |
| 37B | *Phyllophora antartica* |
| 37X | *Phyllophora antartica* |
| 38A | *Phyllophora antartica* |
| 38W | *Phyllophora antartica* |

**b)**

| ITU-CODE | TAXON |
| --- | --- |
| 8C | Filamentous algae (ice core) |
| 9F | *Sterechinus neumayeri* |
| 11D | Filamentous algae (ice core) |
| 11H | *Ophionotus victoriae* |
| 11H | *Sterechinus neumayeri* |
| 11L | *Sterechinus neumayeri* |
| 12B | Filamentous algae (ice core) |
| 12G | *Sterechinus neumayeri* |
| 12I | *Sterechinus neumayeri* |
| 12L | *Sterechinus neumayeri* |
| 13C | Filamentous algae (ice core) |
| 13D | Filamentous algae (ice core) |
|  |  |
|  |  |
|  |  |
|  |  |
| 13H | *Sterechinus neumayeri* |
| 13I | *Sterechinus neumayeri* |
| 13L | *Odontaster validus* |
| 14A | Filamentous algae (ice core) |
| 14B | Filamentous algae (ice core) |
| 14D | Filamentous algae (ice core) |
| 14H | *Sterechinus neumayeri* |
| 14I | *Sterechinus neumayeri* |
| 14L | Amphipoda *(alia)* |
| 14L | *Odontaster validus* |
| 15A | Filamentous algae (ice core) |
| 15G | *Sterechinus neumayeri* |
|  |  |
|  |  |
| 15H | *Sterechinus neumayeri* |
|  |  |
| 15I | *Diplasterias brucei* |
| 15I | *Odontaster validus* |
| 15I | *Ophionotus victoriae* |
| 15I | *Sterechinus neumayeri* |
| 15L | *Odontaster validus* |
| 16A | Filamentous algae (ice core) |
| 16B | Filamentous algae (ice core) |
| 16C | Filamentous algae (interface) |
| 16G | *Aequiyoldia eightsii* |
| 16I | *Diplasterias brucei* |
| 16I | *Ophionotus victoriae* |
| 16I | Serpulidae |
| 16L | *Diplasterias brucei* |
| 16M | *Odontaster validus* |
| 16N | *Odontaster validus* |
| 16P | *Neobuccinum eatoni* |
| 16Q | *Neobuccinum eatoni* |
| 17A | Diatoms (ice core) |
| 17I | *Diplasterias brucei* |
| 17I | *Odontaster validus* |
| 17I | *Ophionotus victoriae* |
| 17L | *Diplasterias brucei* |
| 17L | *Odontaster validus* |
| 17M | *Neobuccinum eatoni* |
| 17N | *Neobuccinum eatoni* |
| 18C | Diatoms (interface) |
| 18E | Benthic diatoms |
| 18E | Diatoms (ice core) |
| 18G | *Ophionotus victoriae* |
| 18H | *Alcyonium antarcticum* |
| 18H | *Ophioplinthus gelida* |
| 18I | *Diplasterias brucei* |
| 18I | *Ophionotus victoriae* |
| 18L | *Urticinopsis antarctica* |
| 18L | *Diplasterias brucei* |
| 18L | *Odontaster validus* |
| 18L | *Ophionotus victoriae* |
| 18M | *Urticinopsis antarctica* |
| 18M | *Neobuccinum eatoni* |
| 18N | *Neobuccinum eatoni* |
| 18N | *Ophioplinthus gelida* |
| 18O | *Neobuccinum eatoni* |
| 19G | Fine-Ultra-Fine Sediment |
| 19H | *Diplasterias brucei* |
| 19H | *Ophioplinthus gelida* |
| 19H | *Ophionotus victoriae* |
| 19I | *Diplasterias brucei* |
| 19I | *Ophionotus victoriae* |
| 19L | *Diplasterias brucei* |
| 19L | *Neobuccinum eatoni* |
| 19L | *Odontaster validus* |
| 19L | *Ophionotus victoriae* |
| 19M | *Urticinopsis antarctica* |
| 19M | *Neobuccinum eatoni* |
| 19M | *Odontaster validus* |
| 19N | *Neobuccinum eatoni* |
| 19W | Diatoms (ice core) |
| 20D | *Iridaea cordata* |
| 20F | Fine Sediment |
| 20H | *Alcyonium antarcticum* |
| 20H | *Diplasterias brucei* |
| 20H | *Perkinsiana* sp. |
| 20I | *Diplasterias brucei* |
| 20I | *Neobuccinum eatoni* |
| 20I | *Ophionotus victoriae* |
| 20I | *Polymastia invaginata* |
| 20L | *Diplasterias brucei* |
| 20L | *Neobuccinum eatoni* |
| 20L | *Odontaster validus* |
| 20L | *Polymastia invaginata* |
| 20M | *Urticinopsis antarctica* |
| 20M | *Odontaster* sp. |
| 20N | *Neobuccinum eatoni* |
| 20P | *Ammothea* sp. |
| 21C | Benthic diatoms |
| 21D | *Sterechinus neumayeri* |
| 21E | *Camptoplites* sp. |
| 21E | *Iridaea cordata* |
| 21E | *Limacina helicina* |
| 21F | *Adamussium colbecki* |
| 21F | Fine Sediment |
| 21H | *Diplasterias brucei* |
| 21H | *Limatula hodgsoni* |
| 21H | *Mycale* sp. |
| 21H | *Ophioplinthus gelida* |
| 21H | Terebellidae |
| 21I | *Adamussium colbecki* |
| 21I | *Diplasterias brucei* |
| 21I | *Odontaster validus* |
| 21I | *Ophionotus victoriae* |
| 21I | *Polymastia invaginata* |
| 21I | *Staurocucumis turqueti* |
| 21L | *Diplasterias brucei* |
| 21L | Nematoda |
| 21L | *Sphaerotylus antarcticus* |
| 21M | *Sphaerotylus antarcticus* |
| 21N | *Adamussium colbecki* |
| 21N | *Sphaerotylus antarcticus* |
| 21O | *Adamussium colbecki* |
| 21O | *Ammothea* sp. |
| 21P | *Ammothea* sp. |
| 21Q | *Ammothea* sp. |
| 21W | Diatoms (ice core) |
| 21W | Ultra-Fine Sediment |
| 22B | *Adamussium colbecki* |
| 22C | *Adamussium colbecki* |
| 22D | *Adamussium colbecki* |
| 22D | Zooplankton |
| 22E | *Adamussium colbecki* |
| 22E | Zooplankton |
| 22E | *Iridaea cordata* |
| 22E | *Limacina helicina* |
| 22E | Fine-Ultra-Fine Sediment |
| 22E | *Staurocucumis turqueti* |
| 22F | Epiphytes |
| 22F | Fine-Ultra-Fine Sediment |
| 22G | *Adamussium colbecki* |
| 22G | *Alcyonium antarcticum* |
| 22G | *Laternula elliptica* |
| 22G | Serpulidae |
| 22G | *Staurocucumis turqueti* |
| 22G | Terebellidae |
| 22H | *Alcyonium antarcticum* |
| 22H | Coarse Sediment |
| 22H | Serpulidae |
| 22H | *Staurocucumis turqueti* |
| 22H | Terebellidae |
| 22I | *Adamussium colbecki* |
| 22I | Nematoda |
| 22I | *Polymastia invaginata* |
| 22I | *Staurocucumis turqueti* |
| 22L | *Adamussium colbecki* |
| 22L | *Colossendeis* sp. |
| 22L | *Colossendeis* sp. |
| 22L | *Odontaster* sp. |
| 22L | *Sphaerotylus antarcticus* |
| 22L | *Staurocucumis turqueti* |
| 22M | *Odontaster validus* |
| 22M | *Sphaerotylus antarcticus* |
| 22N | *Ammothea* sp. |
| 22O | *Ammothea* sp. |
| 22P | *Ammothea* sp. |
| 23A | Diatoms (interface) |
| 23D | Fine-Ultra-Fine Sediment |
| 23E | Phytoplankton |
| 23E | Fine-Ultra-Fine Sediment |
| 23F | *Dendrilla antarctica* |
| 23F | Benthic diatoms |
| 23F | Fine-Ultra-Fine Sediment |
| 23F | Terebellidae |
| 23F | Ascidiacea |
| 23G | *Alcyonium antarcticum* |
| 23G | *Hemigellius* sp. |
| 23G | *Suberites topsenti* |
| 23G | Ascidiacea |
| 23H | Epiphytes |
| 23H | *Staurocucumis turqueti* |
| 23H | Terebellidae |
| 23H | Ascidiacea |
| 23I | *Colossendeis* sp. |
| 23I | *Haliclona* sp. |
| 23I | *Mycale* sp. |
| 23I | Nematoda |
| 23I | *Staurocucumis turqueti* |
| 23L | *Colossendeis* sp. |
| 23L | *Haliclona tenella* |
| 23L | *Sphaerotylus antarcticus* |
| 23M | *Haliclona tenella* |
| 23M | *Hemigellius fimbriatus* |
| 23N | *Achelia* sp. |
| 24A | Diatoms (interface) |
| 24C | Fine-Ultra-Fine Sediment |
| 24E | Epiphytes |
| 24E | Phytoplankton |
| 24E | Fine-Ultra-Fine Sediment |
| 24F | Epiphytes |
| 24G | Epiphytes |
| 24G | *Hemigellius* sp. |
| 24G | *Mycale* sp. |
| 24G | Terebellidae |
| 24H | *Haliclona* sp. |
| 24H | *Ophioplinthus gelida* |
| 24H | Ascidiacea |
| 24I | *Achelia* sp. |
| 24L | *Achelia* sp. |
| 24M | *Suberites topsenti* |
| 24M | *Achelia* sp. |
| 24N | *Suberites topsenti* |
| 24N | *Ammothea* sp. |
| 25E | *Clione limacina antarctica* |
| 25F | *Clione limacina antarctica* |
| 25F | *Diplasterias brucei* |
| 25F | Epiphytes |
| 25G | *Clione limacina antarctica* |
| 25G | Epiphytes |
| 25H | *Perkinsiana* sp. |
| 25H | Zooplankton |
| 25P | Seal Faeces |
| 25Q | Seal Faeces |
| 26E | *Clione limacina antarctica* |
| 26F | *Clione limacina antarctica* |
| 26F | Zooplankton |
| 26H | *Clione limacina antarctica* |
| 26Q | Seal Faeces |
| 26W | Diatoms (interface) |
| 27P | Seal Faeces |
| 28O | Seal Faeces |
| 28Q | Seal Faeces |
| 29D | Phytoplankton |
| 31A | *Phyllophora antarctica* |
| 35B | *Phyllophora antarctica* |
| 35C | *Phyllophora antarctica* |
| 36A | *Phyllophora antarctica* |
| 36B | *Phyllophora antarctica* |
| 36C | *Phyllophora antarctica* |
| 37A | *Phyllophora antarctica* |
| 37B | *Phyllophora antarctica* |
| 37X | *Phyllophora antarctica* |
| 38A | *Phyllophora antarctica* |
| 38W | *Phyllophora antarctica* |

**Table S2.** List of taxa and basal resources found before and after sea-ice breakup in the study area. The top ten central nodes in the food webs (i.e. topological central nodes) are indicated for each web type: ITU, population, and taxonomic. N° is the sampling size (from Sporta Caputi et al., 2020). The ID is the identification code of each food web node shown in Figure 2.

| **Phylum** | **Class** | **Order** | ***Taxon/***  ***Basal resource*** | **Central nodes** | | **N°** | | **ID** |
| --- | --- | --- | --- | --- | --- | --- | --- | --- |
|  |  |  |  | **BEFORE** | **AFTER** | **BEFORE** | **AFTER** |  |
| Anellida | | | |  |  |  |  |  |
|  | Polychaeta | | |  |  |  |  |  |
|  |  | - | *Leitoscoloplos kerguelensis* |  |  | 5 |  | 1 |
|  |  | Phyllodocida | *Barrukia cristata* | ITU-web |  | 7 |  | 2 |
|  |  |  | Hesionidae | ITU-web, population-web |  | 12 |  | 3 |
|  |  |  | Polynoidae | ITU-web |  | 5 |  | 4 |
|  |  |  | *Psamathe fauveli* |  |  | 2 |  | 5 |
|  |  | Sabellida | *Perkinsiana* sp. |  | ITU-web |  | 3 | 6 |
|  |  |  | Serpulidae |  |  | 4 | 3 | 7 |
|  |  | Spionida | Spionidae |  |  | 2 |  | 8 |
|  |  | Terebellida | *Flabelligera mundata* |  |  | 2 |  | 9 |
|  |  |  | Terebellida |  |  | 19 | 7 | 1 |
| Arthropoda | | | |  |  |  |  |  |
|  | Malacostraca | | |  |  |  |  |  |
|  |  | Amphipoda | Amphipoda *(alia)* | Taxonomic-web | Taxonomic-web | 4 | 2 | 11 |
|  |  |  | *Orchomenella* sp. | Population-web, taxonomic-web |  | 6 |  | 12 |
|  |  |  | *Paramoera walkeri* | Taxonomic-web |  | 13 |  | 13 |
|  |  | Isopoda | Arcturidae |  |  | 2 |  | 14 |
|  |  |  | *Munna* sp. | ITU-web |  | 5 |  | 15 |
|  |  |  | Paramunnidae | ITU-web |  | 9 |  | 16 |
|  |  | Tanaidacea | *Nototanais dimorphus* |  |  | 3 |  | 17 |
|  | Pycnogonida |  |  |  |  |  |  |  |
|  |  | Pantopoda | *Achelia* sp. |  |  | 11 | 7 | 18 |
|  |  |  | *Ammothea* sp. | Population-web |  | 23 | 17 | 19 |
|  |  |  | *Colossendeis* sp. |  |  | 2 | 5 | 2 |
|  |  |  | *Nymphon australe* | Population-web |  | 12 |  | 21 |
| Bryozoa | | | |  |  |  |  |  |
|  | Gymnolaemata | | |  |  |  |  |  |
|  |  | Cheilostomatida | *Camptoplites* sp. | ITU-web |  | 8 | 3 | 22 |
| Chordata | | | |  |  |  |  |  |
|  | Ascidiacea | | |  |  |  |  |  |
|  |  |  | Ascidiacea | Population-web |  | 8 | 4 | 23 |
| Cnidaria | | | |  |  |  |  |  |
|  | Anthozoa | | |  |  |  |  |  |
|  |  | Actinaria | *Urticinopsis antarctica* | Taxonomic-web | Taxonomic-web | 7 | 8 | 24 |
|  |  | Alcyonacea | *Alcyonium antarcticum* |  | Population-web | 11 | 9 | 25 |
|  |  |  | *Clavularia frankliniana* |  |  | 3 |  | 26 |
| Echinodermata | | | |  |  |  |  |  |
|  | Asteroidea | | |  |  |  |  |  |
|  |  | Forcipulatida | *Diplasterias brucei* | ITU-web | ITU-web, population-web, taxonomic-web | 22 | 27 | 27 |
|  |  |  | *Diplasteria brucei* (adults) |  |  | 3 |  | 28 |
|  |  | Valvatida | *Acodontaster hodgsoni* | Taxonomic-web |  | 2 |  | 29 |
|  |  |  | *Odontaster* sp. |  | Taxonomic-web | 8 | 3 | 3 |
|  |  |  | *Odontaster validus* | ITU-web, taxonomic-web | ITU-web, population-web | 20 | 22 | 31 |
|  |  |  | *Perknaster fucus antarcticus* |  |  | 2 |  | 32 |
|  | Echinoidea | | |  |  |  |  |  |
|  |  | Camarodonta | *Sterechinus neumayeri* | ITU-web, taxonomic-web | ITU-web, taxonomic-web | 25 | 25 | 33 |
|  | Holothuroidea | | |  |  |  |  |  |
|  |  | Dendrochirotida | *Cucumaria* sp. | Population-web |  | 3 |  | 34 |
|  |  |  | *Staurocucumis turqueti* |  | ITU-web | 14 | 26 | 35 |
|  | Ophiuroidea | | |  |  |  |  |  |
|  |  | Ophiurida | *Ophionotus victoriae* | ITU-web | ITU-web, population-web | 21 | 21 | 36 |
|  |  |  | *Ophioplinthus gelida* | ITU-web | Taxonomic-web | 9 | 6 | 37 |
| Mollusca | | | |  |  |  |  |  |
|  | Bivalvia | | |  |  |  |  |  |
|  |  | - | *Laternula elliptica* | Taxonomic-web |  | 3 | 3 | 38 |
|  |  | Limida | *Limatula hodgsoni* |  |  | 2 |  | 39 |
|  |  | Nuculanida | *Aequiyoldia eightsii* |  |  | 2 | 2 | 4 |
|  |  | Pectinida | *Adamussium colbecki* | ITU-web, population-web, taxonomic-web | ITU-web, population-web, taxonomic-web | 24 | 16 | 41 |
|  | Gastropoda | | |  |  |  |  |  |
|  |  | Neogastropoda | *Neobuccinum eatoni* | Population-web | ITU-web | 24 | 34 | 42 |
|  |  |  | *Trophonella longstaffi* |  |  | 2 |  | 43 |
|  |  | Nudibranchia | *Tritoniella belli* |  |  | 4 |  | 44 |
|  |  | Pteropoda | *Clione limacina antarctica* | Population-web | Population-web | 17 | 9 | 45 |
|  |  |  | *Limacina helicina* |  |  | 50 | 5 | 46 |
| Nematoda | | | |  |  |  |  |  |
|  |  |  | Nematoda |  | ITU-web, population-web, taxonomic-web | 9 | 7 | 47 |
| Nemertea | | | |  |  |  |  |  |
|  | Pilidiophora | | |  |  |  |  |  |
|  |  |  | *Parbolasia corrugatus* | ITU-web |  | 7 |  | 48 |
| Porifera | | | |  |  |  |  |  |
|  | Demospongiae | | |  |  |  |  |  |
|  |  | Dendroceratida | *Dendrilla antarctica* |  |  | 9 | 2 | 49 |
|  |  | Haplosclerida | *Haliclona dancoi* |  |  | 4 |  | 5 |
|  |  |  | *Haliclona* sp. |  |  | 8 | 5 | 51 |
|  |  |  | *Haliclona tenella* |  |  | 2 | 3 | 52 |
|  |  |  | *Hemigellius fimbriatus* |  |  | 3 | 2 | 53 |
|  |  |  | *Hemigellius* sp. |  |  | 7 | 5 | 54 |
|  |  | Poecilosclerida | *Kirkpatrickia variolosa* |  |  | 3 |  | 55 |
|  |  |  | *Mycale* sp. |  |  | 6 | 6 | 56 |
|  |  | Polymastiida | *Polymastia invaginata* |  | ITU-web, population-web | 2 | 4 | 57 |
|  |  |  | *Sphaerotylus antarcticus* | ITU-web | ITU-web, population-web | 6 | 8 | 58 |
|  |  | Suberitida | *Homaxinella balfourensis* |  |  | 5 |  | 59 |
|  |  |  | *Suberites topsenti* | Population-web | Population-web | 10 | 5 | 6 |
| **Food sources** | | |  |  |  |  |  |  |
| Benthic algae | | | |  |  |  |  |  |
|  |  |  | Benthic diatoms | ITU-web |  | 3 | 3 | R1 |
|  |  |  | *Iridaea cordata* | ITU-web |  | 3 | 3 | R7 |
|  |  |  | *Phyllophora antartica* |  |  | 17 | 16 | R14 |
| Epiphytes | | | |  |  |  |  |  |
|  |  |  | Epiphytes |  |  | 6 | 5 | R3 |
| Plankton | | | |  |  |  |  |  |
|  |  |  | Phytoplankton |  |  | 6 | 5 | R8 |
|  |  |  | Zooplankton | Taxonomic-web | ITU-web, taxonomic-web | 4 | 6 | 61 |
| Seal Faeces | | | |  |  |  |  |  |
|  |  |  | Seal Faeces |  |  | 7 | 4 | R4 |
| Sediment | | | |  |  |  |  |  |
|  |  |  | Coarse Sediment |  |  | 3 | 3 | R9 |
|  |  |  | Fine Sediment |  | ITU-web | 3 | 3 | R1 |
|  |  |  | Fine/Ultra-Fine Sediment |  |  | 15 | 6 | R11 |
|  |  |  | Ultra-Fine Sediment |  |  | 3 | 6 | R12 |
| Sympagic algae | | | |  |  |  |  |  |
|  |  |  | Ice core diatoms |  |  | 27 |  | R13 |
|  |  |  | Interface diatoms |  |  | 26 |  | R2 |
|  |  |  | Filamentous Ice core algae |  |  | 27 |  | R5 |
|  |  |  | Filamentous Interface algae |  |  | 20 |  | R6 |

**Table S3.** Detailed contribution (%) of carbon sources to the various sediment fractions, i.e. Coarse (>1 mm), Fine (1-0.75mm), Fine-Ultra-Fine (0.75-0.56 mm) and Ultra-Fine (<0.56 mm), before a) and after b) sea-ice breakup.

**a)**

| **Sediment fraction** | **Carbon source** | **Contribution (%)** |
| --- | --- | --- |
| Coarse | Epiphytes | 9.06 |
| Coarse | Ascidiacea | 9.15 |
| Coarse | *Camptoplites* sp. | 9.27 |
| Coarse | *Haliclona* sp. | 9.15 |
| Coarse | *Hemigellius fimbriatus* | 9.06 |
| Coarse | *Hemigellius* sp. | 9.06 |
| Coarse | Hesionidae | 8.89 |
| Coarse | Nematoda | 8.89 |
| Coarse | *Ophioplinthus gelida* | 9.27 |
| Coarse | Terebellida | 18.21 |
| Fine | Epiphytes | 6.57 |
| Fine | Sympagic algae | 10.18 |
| Fine | *Adamussium colbecki* | 27.47 |
| Fine | Arcturidae | 6.57 |
| Fine | Ascidiacea | 6.57 |
| Fine | *Camptoplites* sp*.* | 17.80 |
| Fine | *Cucumaria* sp. | 6.57 |
| Fine | *Homaxinella balfourensis* | 6.57 |
| Fine | *Orchomenella* sp. | 11.72 |
| Fine-Ultra-Fine | Benthic algae | 10.12 |
| Fine-Ultra-Fine | Epiphytes | 3.01 |
| Fine-Ultra-Fine | Phytoplankton | 5.11 |
| Fine-Ultra-Fine | Sympagic algae | 2.93 |
| Fine-Ultra-Fine | *Adamussium colbecki* | 11.86 |
| Fine-Ultra-Fine | *Alcyonium antarcticum* | 2.72 |
| Fine-Ultra-Fine | Ascidiacea | 1.70 |
| Fine-Ultra-Fine | *Barrukia cristata* | 1.02 |
| Fine-Ultra-Fine | *Camptoplites* sp*.* | 4.13 |
| Fine-Ultra-Fine | *Clione limacina antarctica* | 3.74 |
| Fine-Ultra-Fine | *Cucumaria* sp*.* | 1.97 |
| Fine-Ultra-Fine | *Dendrilla antarctica* | 5.31 |
| Fine-Ultra-Fine | *Diplasterias brucei* | 1.00 |
| Fine-Ultra-Fine | *Homaxinella balfourensis* | 1.02 |
| Fine-Ultra-Fine | *Kirkpatrickia variolosa* | 1.70 |
| Fine-Ultra-Fine | *Laternula elliptica* | 3.09 |
| Fine-Ultra-Fine | *Limacina helicina* | 15.86 |
| Fine-Ultra-Fine | *Mycale* sp*.* | 1.70 |
| Fine-Ultra-Fine | *Nymphon australe* | 2.72 |
| Fine-Ultra-Fine | *Ophionotus victoriae* | 1.00 |
| Fine-Ultra-Fine | *Ophioplinthus gelida* | 1.02 |
| Fine-Ultra-Fine | *Orchomenella* sp*.* | 0.94 |
| Fine-Ultra-Fine | *Paramoera walkeri* | 4.13 |
| Fine-Ultra-Fine | Paramunnidae | 2.41 |
| Fine-Ultra-Fine | *Psamathe fauveli* | 2.16 |
| Fine-Ultra-Fine | Serpulidae | 1.02 |
| Fine-Ultra-Fine | *Staurocucumis turqueti* | 1.70 |
| Fine-Ultra-Fine | *Suberites topsenti* | 1.17 |
| Fine-Ultra-Fine | Terebellida | 3.72 |
| Ultra-Fine | Sympagic algae | 100.00 |

**b)**

| **Sediment fraction** | **Carbon sources** | **Contribution (%)** |
| --- | --- | --- |
| Coarse | Epiphytes | 8.09 |
| Coarse | *Adamussium colbecki* | 8.47 |
| Coarse | *Alcyonium antarcticum* | 8.47 |
| Coarse | *Clione limacina antarctica* | 8.09 |
| Coarse | *Diplasterias brucei* | 8.21 |
| Coarse | *Laternula elliptica* | 8.47 |
| Coarse | *Ophionotus victoriae* | 8.21 |
| Coarse | *Perkinsiana* sp. | 8.30 |
| Coarse | Serpulidae | 8.47 |
| Coarse | *Staurocucumis turqueti* | 8.47 |
| Coarse | Terebellida | 8.47 |
| Coarse | Zooplankton | 8.30 |
| Fine | Benthic algae | 25.05 |
| Fine | Sympagic algae | 7.66 |
| Fine | *Alcyonium antarcticum* | 8.08 |
| Fine | Ascidiacea | 8.25 |
| Fine | *Camptoplites* sp. | 9.15 |
| Fine | *Dendrilla antarctica* | 8.25 |
| Fine | *Diplasterias brucei* | 8.08 |
| Fine | *Limacina helicina* | 9.15 |
| Fine | *Perkinsiana* sp. | 8.08 |
| Fine | Terebellida | 8.25 |
| Fine-Ultra-Fine | Benthic algae | 18.44 |
| Fine-Ultra-Fine | Epiphytes | 6.87 |
| Fine-Ultra-Fine | Phytoplankton | 5.53 |
| Fine-Ultra-Fine | Sympagic algae | 6.62 |
| Fine-Ultra-Fine | *Adamussium colbecki* | 7.29 |
| Fine-Ultra-Fine | *Alcyonium antarcticum* | 6.65 |
| Fine-Ultra-Fine | Ascidiacea | 3.68 |
| Fine-Ultra-Fine | *Camptoplites* sp. | 2.43 |
| Fine-Ultra-Fine | *Dendrilla antarctica* | 3.68 |
| Fine-Ultra-Fine | *Diplasterias brucei* | 1.19 |
| Fine-Ultra-Fine | *Hemigellus* sp. | 1.14 |
| Fine-Ultra-Fine | *Laternula elliptica* | 3.20 |
| Fine-Ultra-Fine | *Limacina helicina* | 4.15 |
| Fine-Ultra-Fine | *Mycale* sp. | 1.14 |
| Fine-Ultra-Fine | *Ophionotus victoriae* | 0.89 |
| Fine-Ultra-Fine | *Ophioplinthus gelida* | 2.25 |
| Fine-Ultra-Fine | *Perkinsiana* sp. | 3.48 |
| Fine-Ultra-Fine | Serpulidae | 3.20 |
| Fine-Ultra-Fine | *Staurocucumis turqueti* | 4.92 |
| Fine-Ultra-Fine | Terebellida | 8.02 |
| Fine-Ultra-Fine | Zooplankton | 5.23 |
| Ultra-Fine | Phytoplankton | 23.82 |
| Ultra-Fine | Sympagic algae | 76.18 |
